# A qualitative exploration of the psychosocial factors of sexual satisfaction in Iranian newlyweds

**DOI:** 10.1371/journal.pone.0356277

**Published:** 2026-08-13

**Authors:** Zahra Toghiyani, Ashraf Kazemi, Mahbobeh Taebi, Fatemeh Mohammady

**Affiliations:** 1 Student research committee, School of Nursing and Midwifery, Isfahan University of Medical Sciences, Isfahan, Iran; 2 Nursing and Midwifery Care Research Center, Isfahan University of Medical Sciences, Isfahan, Iran; Tarbiat Modares University Faculty of Medical Sciences, IRAN, ISLAMIC REPUBLIC OF

## Abstract

**Background:**

Psychosocial factors related to sexuality, such as gender expectations and interpersonal dynamics, influence sexual satisfaction, and identifying these factors is beneficial for developing educational programs for newly married couples. However, evidence regarding these psychosocial factors in the Iranian sociocultural context is limited. Therefore, the present study was conducted to identify the psychosocial factors influencing sexual satisfaction among newly married couples.

**Method:**

This study employed a qualitative content analysis approach. It involved 31 heterosexual newly married individuals who had been sexually active for less than two years. Purposive sampling was used and continued until data saturation was achieved. Data were collected through 36 semi-structured interviews, including 31 individual interviews and 5 couple interviews, and were analyzed using qualitative content analysis. The reporting of this qualitative study adhered to the Consolidated Criteria for Reporting Qualitative Research guidelines.

**Results:**

Premarital sexual norms, sexual schemas, and sexual agency and control. were the three main psychosocial categories identified through the qualitative analysis. Social norms reflected taboos on sexual self-disclosure, social norms of restraint sexual desires, and the obligation to preserve virginity in women. Sexual schemas encompassed sexual fantasies, attitudes toward sexual issues, and perceived sexual competence, while sexual agency and control involved sexual awareness and skills, sex as a means of conflict resolution, and controlling sex.

**Conclusion:**

The findings provide insight into the key psychosocial dimensions underlying sexual satisfaction among newly married couples in Iran. These findings provide a culturally grounded understanding of how social norms, sexual schemas, and sexual agency shape sexual experiences and may inform the development of culturally sensitive sexual education, counseling, and intervention programs aimed at promoting sexual well-being and marital adjustment.

## Introduction

Sexual satisfaction reflects an individual’s cognitive and emotional evaluation of the extent to which their sexual needs and expectations are fulfilled [1]. As a key component of sexual health, it contributes to couples’ quality of life by enhancing marital satisfaction [2] and strengthening marital relationships [3]. Since sexual relationships are shaped by sociocultural, legal, and religious norms, understanding the factors associated with sexual satisfaction is particularly important in contexts where intimate and sexual relationships are recognized only within marriage, especially during the early years of marriage.

This period represents a critical developmental stage during which couples establish and consolidate patterns of intimacy, communication, conflict management, and dyadic adjustment that may shape subsequent marital quality, sexual satisfaction, and relationship stability [4,5]. Difficulties in achieving and maintaining sexual satisfaction during this formative stage may have far-reaching consequences. Decreased marital and sexual satisfaction has been associated with depression [6], lower self-esteem [7], emotional divorce [8], and marital instability, as well as social consequences such as extramarital sexual relationships [9].

The adverse consequences of sexual dissatisfaction have prompted increasing research attention toward identifying the determinants of sexual satisfaction in diverse cultural and social contexts, particularly among newly married couples navigating the transition to married life. Understanding these determinants can inform the design and implementation of targeted sexual health counseling interventions and premarital education programs. The significance of this transitional period may be even greater in societies such as Iran, where sociocultural and religious norms often restrict premarital sexual experiences and limit open communication about sexual matters [10].

From a biopsychosocial perspective, sexual satisfaction is a multidimensional construct that extends beyond biological functioning, including sexual desire, arousal, and physiological sexual responses, and is shaped by dynamic interactions among psychological, interpersonal, and sociocultural factors [11–13]. Sociocultural factors related to sexuality within a society play a significant role in shaping individuals’ expectations, beliefs, and perceptions of sexual relationships even before they enter into sexual experiences [12].

In such societies, it is necessary to conduct an in-depth exploration of attitudes related to sexual satisfaction among newlyweds based on their lived experiences, since these attitudes are not formed in isolation but are influenced by interacting psychological and social factors. Psychosocial factors refer to the dynamic interaction between social influences, such as family relationships, social support, and cultural norms, and psychological processes, such as beliefs, attitudes, and emotions, which shape individuals’ perceptions, behaviors, and overall well-being [14].

In Iran, cultural and social discourses, reinforced through family, education, and policy, contribute to a social reality in which sexuality is largely privatized and moralized. Empirical studies confirm that taboos are reflected in parents’ conservative approaches to sexuality education for their children [15], as well as in the reluctance or inability of engaged or newly married couples to obtain accurate information or express mutual sexual needs. For instance, a qualitative study of engaged couples in Iran showed that sociocultural constraints and inadequate knowledge are key challenges [16].

Conducting research in this area is essential not only for advancing scientific knowledge but also for providing culturally sensitive insights that can inform counseling, educational programs, and interventions aimed at strengthening marital relationships. By identifying the key psychosocial predictors of sexual satisfaction among newlywed Iranian couples, researchers and practitioners can contribute to promoting healthier marriages, reducing marital conflicts, and enhancing the overall quality of family life.

The ongoing sociocultural transition from traditional to more modern value systems has created unique psychosocial conditions that shape expectations regarding marriage, intimacy, and gender roles. These conditions may be particularly influential for newly married couples who are navigating the early years of marriage, a period characterized by ongoing sexual and relational development. The coexistence of traditional beliefs and modern attitudes may create challenges, tensions, and opportunities that influence couples’ experiences of sexual satisfaction. However, little is known about how newly married couples perceive and experience these influences within their specific cultural and social context. Given this knowledge gap and the context-dependent nature of sexual satisfaction, a qualitative approach is warranted to gain an in-depth understanding of the determinants of sexual satisfaction from the perspectives of newly married couples. The qualitative research allows for the examination and interpretation of how individuals interact with different phenomena within their specific social context [17], this approach is appropriate for deeply understanding the psychosocial factors related to sexual satisfaction. Accordingly, this study aimed to identify the psychosocial factors of sexual satisfaction among Iranian newlywed couples, and the research question was: “What are the psychosocial factors of sexual satisfaction in newly married couples?”

## Methods and materials

This qualitative study was conducted in Isfahan, Iran, with the approval of the Ethics Committee of Isfahan University of Medical Sciences (IR.MUI.NUREMA.REC.1401.130). The study was reported in accordance with the Consolidated Criteria for Reporting Qualitative Research (COREQ) guidelines (Supplement 1). All methods were performed in accordance with the relevant guidelines and regulations. An inductive qualitative approach was chosen to explore psychosocial factors of marital sexual satisfaction because it allows themes and concepts to emerge directly from couples’ lived experiences, without being constrained by pre-existing theories. Given the sensitive and context-dependent nature of sexual satisfaction, this approach increases the likelihood of uncovering novel, culturally specific, and nuanced psychosocial variables. It supports developing a rich, authentic understanding grounded in the data, enhancing credibility and relevance of findings in the study’s specific social context [18].

### Participants

The participants in this study included 31 heterosexual Iranian women and men, all of whom were Persian-speaking, aged 19–44 years, had at least a high school diploma, and had been sexually active with their spouses for less than two years. The characteristics of the participants are presented in Table 1.

**Table 1:** Individual characteristics of the participants.

Number	Age	Gender	Educational level	Duration of marriage (month)
1	25	Woman	Bachelor degree	36
2	33	Man	Master degree	60
3	28	Woman	Bachelor degree	7
4	25	Woman	Bachelor degree	9
5	21	Woman	Diploma	9
6	32	Man	Diploma	12
7	28	Man	Bachelor degree	5
8	19	Woman	Diploma	12
9	24	Woman	Bachelor degree	12
10	33	Woman	Bachelor degree	15
11	37	Woman	Bachelor degree	16
12	24	Woman	Bachelor degree	5
13	28	Woman	Master degree	17
14	33	Woman	Philosophy doctor	60
15	32	Woman	Bachelor degree	12
16	33	Man	Philosophy doctor	12
17	30	Woman	Bachelor degree	13
18	27	Man	Master degree	12
19	24	Woman	Master degree	48
20	32	Woman	Master degree	24
21	32	Man	Master degree	48
22	32	Woman	Master degree	36
23	34	Woman	Bachelor degree	48
24	37	Woman	Bachelor degree	12
25	44	Man	High school	15
26	34	Woman	Diploma	15
27	31	Man	Diploma	60
28	38	Man	Bachelor degree	48
29	31	Man	Bachelor degree	24
30	26	Man	Diploma	12
31	38	Man	Diploma	24

The inclusion criteria included having no known mental and behavioral disorders or chronic physical diseases affecting sexual relations. Also, given the potential impact of pregnancy and infertility on sexual satisfaction, these conditions were considered exclusion criteria. As some couples in Iran may delay cohabitation and the initiation of sexual activity following official marriage registration, individuals whose marriages had been formally registered for more than five years were excluded from the study.

Participants were recruited among clients attending premarital education centers, health service centers, and sexual counseling centers. In Iran, all couples are required to participate in premarital counseling before marriage, and after marriage they have a health record in a city health center. Therefore, these centers offer access to the target population and enable sampling with maximum diversity. Participants were purposively selected to ensure variation in characteristics such as age, sex, duration of marriage, educational level, employment status, and socioeconomic background, thereby capturing a broad range of experiences and perspectives related to sexual satisfaction.

### Procedure and data collection

After introducing the researcher and explaining the study’s purpose, eligibility criteria were evaluated. For those who met the inclusion criteria, the research process, ethical considerations, and their rights as participants were explained and written consent was obtained from all eligible individuals who accepted the invitation to take part in the study. The timing and location of each interview were arranged by mutual agreement between researcher and participant. All interviews took place in a private room at the centers. Interview scheduling was based on the participants’ preferences.

Data were collected through face-to-face, semi-structured, in-depth interviews using a voice recorder. Interviews were conducted by a female member of the research team who was a PhD student in reproductive health, under the supervision of a reproductive health specialist. Each participant was first interviewed individually, followed by a dyadic interview with their spouse. Individual interviews provided a private setting in which participants could freely discuss personal and potentially sensitive experiences. The subsequent dyadic interviews offered opportunities to explore shared experiences, relational dynamics, and the ways couples jointly negotiated and constructed meanings around their sexual relationship. We acknowledge that the presence of a partner during dyadic interviews may have influenced participants’ disclosure or expressions of disagreement. These potential influences were considered during data interpretation by comparing the accounts obtained from the individual and dyadic interviews. All interviews were conducted in a conversational manner to foster trust and encourage rich, nuanced accounts. Data collection continued until no new codes or concepts emerged from successive interviews, indicating that data saturation had been achieved.

Open-ended guiding questions were developed to ensure coverage of the main research objectives, while allowing participants the freedom to elaborate and introduce new themes. The guiding questions were developed based on three unstructured interviews, whose data were not used in the study’s data analysis. Based on these interviews, a set of guiding questions was developed. Examples include:

- In your social interactions with family members and others, what kinds of understandings about sexuality have been formed for you?
- If you wished to discuss sexual matters with your close relatives, what kinds of behaviors would you expect to observe?
- When you experience sexual needs or desires, how do you usually respond or manage them?
- In what ways did your family, cultural background, or upbringing shape your attitudes toward sexuality and sexual satisfaction?

Moreover, probing questions, such as “Tell us about your feelings at the time” and “What was your impression” were used to deepen the interviews. The interviewer also documented non-verbal expressions, including body movements and gestures that conveyed participants’ emotions during the interviews, as part of the qualitative data collection process. In order to prevent the bias of the spouse’s presence in couple interviews, an attempt was made to deepen the interviews with questions such as “Could you elaborate on what your spouse has just said?”

The individual interviews lasted between 50 and 70 minutes, while the couple interviews ranged from 60 to 85 minutes in duration. A total of 36 semi-structured interviews were conducted. First, 31 individual interviews were conducted with 19 women and 12 men. Subsequently, 5 interviews were conducted with 5 couples (10 participants), all of whom had previously participated in the individual interviews.

### Data analysis

Manual analysis of the data was carried out using Graneheim and Lundman’s method [19] in parallel with sampling. Shortly after each interview, the audio file was listened to several times and was transcribed verbatim by the first author. Previously recorded observations were noted next to the narrations. The first and second authors read the transcribed text and field notes several times to ensure a thorough understanding of the content.

Meaning units were identified as words, sentences, or paragraphs that related to the same central meaning. Each meaning unit was condensed, i.e., shortened while preserving the core meaning, and inferential codes were then elicited from them. Subsequently, the codes were compared based on similarities and differences and grouped into subcategories, secondary categories, and overarching categories through an iterative process of abstraction. The categories were therefore derived inductively from the participants’ narratives rather than being predefined by existing theoretical frameworks. After the categories had emerged, relevant theoretical concepts from the literature were consulted to support their conceptual interpretation and naming, where appropriate. (Table 2).

**Table 2:** Example of data analysis.

Category	Sub-category	Code	Condensed Meaning Unit	Meaning Unit
Premarital sexual norms	Obligation to preserve virginity in women	Stigma of breaking the hymen before marriage	The disaster of breaking one’s virginity before marriage and the obligation to take care of it.	Breaking the hymen before marriage, for whatever reason, is a disaster. Since childhood, my mother always reminded me to be careful not to harm my hymen while playing. If my hymen were broken (when I was single), everyone would think that I had a relationship with someone.

Interview transcripts of the joint interviews were analyzed using thematic analysis with individual-level coding. Each partner’s responses were first coded separately to capture unique perspectives and experiences. Subsequently, a comparative process was undertaken to examine convergence and divergence between partners’ accounts. This two-step approach allowed for the identification of both individual meanings and relational dynamics, highlighting how sexual satisfaction is perceived, negotiated, and co-constructed within the couple. Analytical memos were used to document observations of interactional patterns and contextual factors emerging from the dyadic setting, thereby ensuring a comprehensive interpretation of the psychosocial influences on sexual satisfaction.

### Trustworthiness

In order to ensure the accuracy and rigor of the data, four criteria of credibility, dependability, transferability, and confirmability were used [20]. To enhance the credibility of the findings, an audit trail of the reduction process was maintained in a coding matrix, documenting how meaning units, condensed text, codes, and categories were linked. Research team members independently coded portions of the data and then met to discuss discrepancies until consensus was achieved. Peer debriefing and iterative discussions ensured the stability and trustworthiness of the final categorization.

Also, encompassed external peer review, long-term engagement with the participants, and participant review strategies were implemented. To ensure the adaptability of the data, the experts’ opinions were used. Also, the dependability of the data was strengthened through a comprehensive description of each research stage. To enhance the transferability of the data, the research findings were made available to three new couples for assessment regarding the alignment between the findings and their own experiences. To enhance the confirmability of the data, three interviews were reviewed by four external experts in qualitative research, two specializing in reproductive health and two in psychology. These experts were tasked with verifying the accuracy of the coding process.

## Results

Of the 34 eligible individuals invited to participate, 31 (19 women and 12 men) completed individual interviews. Three eligible individuals declined to participate. The participants included 19 women and 12 men with the mean age of 29.5 (SD: 5.32) and 32.75 (SD: 5.13) years respectively. The characteristics of the participants are presented in Table 1.

The analysis of the participants’ experiences led to the extraction of 720 inferential codes. After merging similar codes, the number of codes was reduced to 234. The extraction of three main categories of “Premarital sexual norms,” “Sexual schemas,” and “Sexual agency and control” was the result of analyzing the data in the study population (Table 3).

**Table 3:** Main and subcategories.

Main Categories	Subcategories	Main codes
Premarital sexual norms	Taboo of sexual self-disclosure	Embarrassment about obtaining sexual information
		Perceiving the sinfulness of sexual needs
		Embarrassment about sexual demands
	Social norms of restraint sexual desires	Sinfulness of sexual thoughts
		Sinfulness of masturbation
		Value of sexual restraint
	Obligation to preserve virginity in women	Stigma of breaking the hymen before marriage
		Requirement of confirming virginity in sexual relations after marriage
		Feeling of being sexually threatened by men
Sexual schemas	Sexual fantasies	Sexual diversification
		Sexual fantasizing
		Seeking affection versus pleasure
	Attitude towards sex	Attitudes toward sex
		Sexual expectations
		Sexual obligation
	Perceived sexual competence	Sexual attractiveness
		Interactive sexual behavior
		Playing sexual roles
Sexual agency and control	Interactive sexual behavior and sexual awareness	Sexual awareness
		Skill of expressing sexual needs and demands
		Skill of satisfying sexual needs
	Sex as a means of conflict resolution	Means of resolving marital conflicts
		Prevention of extramarital relations
		Maintaining marital intimacy
		Sexual authoritarianism: a means of confirming authority
	Mutual understanding in sex	Activism versus passivity
		Mutual understanding of sexual emotions
		Active interaction in a sexual relationship

### 1. Premarital sexual norms

This category was derived from three subcategories: ‘the taboo of sexual self-disclosure before marriage,’ ‘social norms of restraint sexual desires before marriage,’ and ‘obligation to preserve virginity in women before marriage.’

#### 1.1. The taboo of sexual self-disclosure before marriage.

The subcategory, ‘the taboo of sexual self-disclosure before marriage,’ was inferred from three main codes: ‘embarrassment about obtaining sexual information,’ ‘perceiving the sinfulness of sexual needs,’ and ‘embarrassment about sexual demands.’ These main codes were extracted from participants’ statements and indicated the taboo of social issues in society that caused the participants to feel embarrassed to express their need for sexual information.

Moreover, the taboo of expressing sexual issues made some participants, especially during adolescence, feel guilty about their sexual emotions and embarrassed about expressing their need to satisfy their sexual desires. One of the male participants (P2) stated: “*At the beginning of my married life, I felt there was a lot that I needed to know about sexual issues. But in our family, we weren’t allowed to discuss such issues. Once my uncle said something about AIDS, my father got up and said angrily: “Shame on you. You shouldn’t talk about these things in public; family is respected. I didn’t even think about asking my Dad about sex. It’s shameful”*

#### 1.2. Social norms of restraint sexual desires before marriage.

The category of premarital sexual norms was derived from the three main codes of ‘sinfulness of sexual thoughts,’ ‘sinfulness of masturbation,’ and ‘the value of sexual restraint.’

The sinfulness of sexual thoughts was inferred from the statements of the participants who said that they felt guilty by thinking about sexual topics and remembering the sexual scenes that they had seen willingly or unwillingly and tried to get rid of those thoughts. They stated that sometimes they would satisfy their sexual needs by having sexual thoughts in their privacy or by masturbating; however, they were ashamed and felt guilty about it. In this regard, the woman (P15) stated: *“When I was obsessed with sexual thoughts, it was then beyond my control. I felt that the devil was roaming around and pushing me through these thoughts; I felt helpless. After that, I asked God to forgive me.”*

The value of sexual restraint was also extracted from statements showing that individuals were in a social context where sexual abstinence and suppression of sexual needs were considered social value. This social value was formed by establishing social messages that reflected the standard of perfection in women and men as overlooking sexual needs. In this regard, a man (P16) stated: *“I was involved (in sexual excitement) before marriage. …., but I couldn’t help it. “I had to suppress it; otherwise, it would mislead me. If I gave in, I’d lose control and be unable to resist. I’d end up ensnared by it, whether through masturbation or other shameful acts.”*

#### 1.3. Obligation to preserve virginity before marriage.

Obligation to preserve virginity was one of the subcategories from which the category of premarital sexual norms was extracted. This sub category was derived from the main codes of ‘the stigma of breaking the hymen before marriage,’ ‘the requirement of confirming virginity in sexual relations after marriage,’ and ‘the feeling of being sexually threatened by men.’

The perception of the stigma of breaking the hymen before marriage was extracted from experiences showing that the female participants received messages since their childhood that breaking the hymen was associated with negative social judgments about girls and their families. Therefore, preventing it from breaking before marriage was considered a social duty for girls and family members, and breaking the hymen during the first intercourse with the husband indicated the chastity of the woman. The woman (P9) expressed her experiences in this regard as follows: *“Breaking the hymen before marriage, for whatever reason, is a disaster. Since childhood, my mother always reminded me to be careful not to harm my hymen while playing. If my hymen were broken (when I was single), everyone would think that I had a relationship with someone or that I was raped. I wasn’t considered a virgin.”*

The analysis of the interviews showed that the social value of virginity and the commitment to preserve virginity had caused men to be perceived as potential aggressors, and in order to foil this threat, women were obliged to avoid the possibility of harm in regular social relationships and interactions. Based on these experiences, ‘Feeling of being sexually threatened by men’ was deduced. For instance, a woman (P8) said: *“Since I was a child, the family didn’t allow me to go alone where there were men or boys, even to relatives’ houses. One day, I was playing outside the house with a boy. My brother came and saw me. He took my hand and took me home. He said: “Didn’t I tell you not to play with boys?” He also told my mom not to let me play outside anymore. A mishap (sexual rape) would happen to me, which would cause disgrace. Because of that, I was always afraid of men.”*

### 2. Sexual schemas

Sexual schemas were inferred from the subcategories: ‘sexual fantasies,’ ‘attitudes towards sex,’ and ‘perceived sexual competence.’

#### 2.1. Sexual fantasies.

Sexual fantasies were extracted from the main codes: ‘sexual diversification,’ ‘sexual fantasizing,’ and ‘seeking affection versus pleasure.’

Sexual diversification was extracted from narratives indicating that a pleasurable sexual relationship was not necessarily defined as penile-vaginal intercourse. Some participants recognized the desire for sex in a variety of ways as a measure of pleasurable sex and molded their sexual dreams accordingly.

One of the male participants (P6) stated, “*Before marriage, I imagined sex differently, just like in erotic movies. I would have sex in diverse ways in my sexual imagination. I even thought that the orgasm would be much more enjoyable than this*.” When asked how this perception of sex has affected your sex life, this participant replied, “*Our sex is not what I thought it would be; repeated sex; I’m satisfied, and that’s it*.”

In this regard, a woman (P14) stated that *“My husband says that he doesn’t enjoy sex with me anymore. It’s boring. He expects me to do the dirty things that the pornographic actresses do.”*

#### 2.2. Attitude toward sex.

Attitudes toward sexual issues were inferred from: ‘Attitudes toward sex,’ ‘Sexual expectations,’ and ‘Sexual obligation. ‘Attitudes towards sex’ was concluded from narratives that reflected individuals’ approach about sexual issues. One of the female participants (P3) expressed her experiences: *“I have a traditional family, and we can’t talk about these issues in our company. Once, I said to my mother what the schoolchildren had said about male-female sexual relations. My mother said: “These are bad words. I don’t want to hear you talk about them again.”*

The main code ‘sexual expectancies’ refers to the prospects the participant expected from the sexual relationship. Some participants considered the outcome of sex an essential and valuable element, and others did not value it. This code refers to the participants’ expectations of ideal sex. Some expected to have penile-vaginal intercourse, and some considered reaching orgasm in sexual intercourse as the main goal of sexual intercourse, and they expected their spouse to care about this issue during sexual intercourse. In this regard, a woman (P1) said: *“Sexual relationship is a part of marital life. I don’t care how it passes.*

Sexual obedience was another main code that shaped the attitude toward sexual issues. This subcategory mentioned the gender attitude that the participants had toward their and their spouse’s sexual duties. Some believed that women had the duty to satisfy their husband’s sexual desires. Some others believed that both of them were required to respect each other’s sexual desires during sex and make an attempt to fulfill them. In this regard, a woman participant (P11) stated: *“In our* *married life, sometimes I don’t want to have sex. But I do it because every woman is obliged to (sexually) satisfy her husband. I don’t feel good about this (sexual) relationship, but I am satisfied because I am fulfilling my duty.”*

#### 2.3. Perceived sexual competence.

The subcategory of ‘perceived sexual competence’ was extracted from the main codes of ‘sexual attractiveness,’ ‘Interactive sexual behavior,’ and ‘playing sexual roles.’

These main codes were extracted from narratives indicating that in order to have an ideal sexual relationship, the adaptation of behaviors and the attractiveness of the sexual organs and even the shape of the body with the social criteria of attractiveness, as well as the requirement to have the skill of sexual behaviors in some cases, were required.

One of the female participants (P8), who considered her lack of sexual attractiveness as the reason for her husband’s low sexual satisfaction, said: *“Who can be excited by this thin body with small breasts? My husband likes my breasts to be uplifted. Maybe in that case, my husband would like to have more sex.”*

Another participant (P20) said, “*My husband doesn’t know how to satisfy me. When I object to him, he expects me to try it myself. He must know how to have intercourse so that I can be satisfied.*” This participant continued, “*How would I know that? Men easily talk about sexual issues and learn from each other. But learning these things (sex) is against the custom for women, especially before marriage*.”

### 3. Sexual agency and control

Sexual agency and control was another category extracted from the data and the three subcategories of ‘sexual knowledge and skills,’ ‘sex as a means of control,’ and ‘controlling sex.’

#### 3.1. Interactive sexual behavior and sexual awareness.

The subcategory of sexual awareness and skill was derived from the main codes of ‘Sexual awareness,’ ‘the skill of expressing sexual needs and demands,’ and ‘the skill of satisfying sexual needs.’ Regarding the sexual awareness, some participants stated that they lacked the necessary knowledge to be in sexual relations due to social and cultural barriers before marriage. In order to have an ideal sex life, they felt it necessary to gain more knowledge about their sexual issues. In this regard, a man participant (P25) said: *“My wife complains that she doesn’t have an orgasm in sex. She believes I don’t know how to satisfy her. I tell her that she herself should know what to do to have an orgasm. Everyone should learn how to enjoy their relationship (sexual relationship).”*

In addition to sexual awareness, the skill of expressing sexual needs and demands was another main code of sexual awareness and skills subset. This code was specifically extracted from the analysis of sexual experiences of women who stated that due to the taboo of sexual issues, they had not been able to acquire skills in expressing their needs and demands and believed that their unexpressed sexual needs should be met by their spouse. However, men mainly believed their wives needed to express their needs and demands. In this regard, a woman participant (P26) stated: *“Many times, I desire to have sex, but I can’t tell my husband directly because I’m embarrassed. I don’t even know how to show that I need it with my behavior. When I complain about it, he says that he’s not a mind reader and that I should somehow say or at least show it. But I think he should figure it out.”*

The skill of satisfying sexual needs was another subcategory that formed the main codes of sexual awareness and skills. The analysis of the participants’ experiences showed that some individuals believed that in order to have pleasurable sex, both partners needed to have the skills to meet their sexual needs and be actively involved in this relationship. Some others believed that the sexual partner needed to have enough skills to meet their spouse’s sexual needs. In this regard, a woman (P22) stated: *“My husband says that I should know how to do it myself to be able to achieve sexual pleasure. He says that he doesn’t have any problems, and he knows how to satisfy himself.”*

#### 3.2. Sex as a means of conflict resolution.

The subcategory of ‘Sex as a means of conflict resolution’ was extracted from the main codes of ‘Means of resolving marital conflicts,’ ‘prevention of extramarital relations,’ ‘maintaining marital intimacy,’ and ‘sexual authoritarianism: a means of confirming authority.’

The main codes of ‘Means of resolving marital conflicts’ was extracted from experiences indicating that some sexual partners had sexual intercourse to establish a new emotional relationship or to appease the spouse after marital conflicts or disputes. In this regard, a woman participant (P23) who believed her husband lacked proper understanding of the cause of the conflict said: *“When we have an argument, my husband has sex that night. He believes that everything will be solved this way. “Whenever he wants our relationship to feel intimate, he initiates sex, so that he doesn’t have to respond to many other issues.”*

Another main code that shaped ‘Sex as a means of conflict resolution was prevention of extramarital relations. This subcategory was extracted from the inferential codes showing that some women considered sex with their spouse as a means to prevent extramarital affairs by their husband. In this regard, a woman (P19) said: *“Men have higher (sexual) needs than women. He will go out with someone else if I don’t meet them. I have to accept sex to preserve my married life, even if I don’t like it or I’m ready for it.”*

By analyzing the experiences of some participants, the main code ‘maintaining marital intimacy’ was extracted, which showed that for some individuals, sex was a way to achieve and maintain intimacy with their spouse. In this regard, a woman (P3) said: *“When we have sex, I feel that I love him more, and he loves me more, and I can trust him more. This shows that he still loves me.”*

The subcategory sexual authoritarianism; a means of confirming authority’ refers to the experiences of participants who expressed that despite their reluctance to have sex, their husbands tried to show their authority in their lives by having sex. In this regard, a woman (P1) said: *“Sexual intercourse that is meant to show dominance is not satisfactory. Despite my unwillingness, my husband has sex with me to show that what he says goes.”*

#### 3.3. Mutual understanding in sex.

Another subcategory shaping sexual agency was ‘mutual understanding in sex,’ which was extracted from the main codes of ‘activism versus passivity’, ‘mutual understanding of sexual emotions,’ and ‘active interaction in a sexual relationship.’

The main codes main cod of ‘activism versus passivity’ was derived from the statements that couples made regarding their sexual relationship. The analysis of the data showed that men actively participated in sex, while women had sex passively in response to men’s sexual behavior. In this regard, a man participant (P16) stated: *“I always do something (sexual behaviors); she waits for me to do it to make her satisfied. In sex, women are more passive, and men are active. Sex is a masculine skill.”*

The ‘Mutual understanding of sexual emotions was derived from the statements of the participants indicating that, in a sexually satisfying relationship, they needed to understand their spouse’s sexual reactions. The main cod of ‘active interaction in a sexual relationship’ was inferred from the participants’ statements, indicating that mutual active participation was a significant factor in a satisfactory sexual relationship. For instance, a man (P18) stated: *“My wife shows no reaction during sex. I like her to show it if my stimulations cause her sexual pleasure. This way, I will be more excited and have more sexual pleasure. She must do something during sex.”*

## Discussion

This qualitative study aimed to explore the psychosocial determinants of sexual satisfaction among Iranian newlyweds. The findings reveal that psychosocial factors, particularly social norms, sexual schemas, and sexual agency, play a critical role in shaping sexual satisfaction during the early years of marriage. Participants’ accounts further suggest that these psychosocial factors are closely interconnected, with prevailing social norms shaping individuals’ sexual schemas, which may in turn influence the development and expression of sexual agency, thereby contributing to experiences of sexual satisfaction.

The psychosocial factors identified in the present study appear to influence sexual satisfaction through their impact on the cognitive, emotional, and behavioral dynamics of intimate relationships. Social norms constitute a sociocultural framework that shapes individuals’ sexual attitudes, expectations, and relational behaviors, whereas sexual schemas function as cognitive frameworks through which sexual experiences are interpreted and sexual self-concepts are constructed. In addition, sexual agency facilitates individuals’ ability to communicate preferences, negotiate desires, and engage in sexual interactions with confidence and autonomy.

Incongruence between partners’ sexual norms, schemas, and expectations may negatively affect sexual satisfaction by shaping divergent perceptions and interpretations of sexual experiences. Such discrepancies can lead to misunderstandings, communication difficulties, and unmet sexual expectations, thereby undermining emotional intimacy and mutual responsiveness. Consequently, couples may experience lower levels of sexual satisfaction, particularly during the early years of marriage when shared meanings and patterns of sexual interaction are still being established [21].

This interpretation is consistent with social constructionist perspectives, which propose that sexual experiences are not merely individual or biological phenomena but are shaped through dominant cultural narratives, religious traditions, and gendered expectations [22]. Within this framework, couples’ sexual expectations and behaviors are understood to be influenced by socially constructed meanings, which may contribute to discrepancies in sexual norms and schemas and, consequently, to lower sexual satisfaction.

A key theme was the persistence of social taboos surrounding sexuality. From childhood through adulthood, participants internalized prohibitions against premarital sexual expression, resulting in feelings of guilt, shame, and anxiety regarding sexual thoughts, desires, and behaviors. This taboo was strongly associated with restricted communication, lack of reliable sexual knowledge, and unrealistic marital expectations, thereby reducing opportunities for intimacy and satisfaction. The findings suggest that, within the study population, prevailing social norms positioned virginity as both a gendered role and a social obligation for women. This emphasis on the preservation of virginity may underpin broader taboos surrounding sexual matters, contributing to restrictions on accessing sexual information and inhibiting the open expression of sexual needs. The cultural emphasis on female virginity prior to marriage further reinforced restrictive norms, framing women’s sexual purity as a symbol of family honor and positioning men as potential threats to this purity. The psychosocial factors identified in the present study, particularly social norms, are consistent with Sexual Script Theory, which posits that societal narratives shape acceptable sexual roles by granting men greater sexual agency while encouraging women to embody modesty, passivity, and compliance [23]. The findings of the present study are consistent with evidence from countries characterized by conservative religious and sociocultural norms regarding sexuality and sexual health. Studies conducted in South Ethiopia, Bangladesh, and India have demonstrated that sexual taboos and limited openness in discussing sexual matters may hinder individuals’ access to reliable sexual health information [24,25]. But even in countries such as Australia, research has demonstrated that sexual shame and embarrassment can reduce individuals’ willingness to seek accurate sexual information from credible and professional sources [26].

Together, these findings suggest that while the influence of restrictive social norms and sexual taboos may be particularly pronounced in conservative sociocultural contexts such as Iran, the adverse effects of sexual shame on seeking sexual information appear to extend across diverse cultural settings. This comparison indicates that some psychosocial processes underlying sexual satisfaction may be broadly shared, whereas their expression and intensity are shaped by the surrounding sociocultural context.

Another finding of the study was the concept of sexual schema, which emerged from the subcategories of sexual fantasies, attitudes toward sexual issues, and perceived sexual competence. Based on a systematic review of studies conducted in the United States, sexual schemas are conceptualized as a multidimensional construct encompassing individuals’ cognitive representations, beliefs, expectations, and assumptions regarding sexuality [27]. This conceptualization is consistent with the findings of the present study, which likewise identified these dimensions as central components of sexual schemas.

Importantly, analysis of this concept revealed notable gender differences in sexual schemas. Women prioritized emotional intimacy in their sexual experiences and reported dissatisfaction when such intimacy was absent, whereas men were more oriented toward physical pleasure and variety. These mismatches in expectations illustrate the influence of gendered sexual schemas on marital satisfaction. Findings of the present study are consistent with prior research indicating that gendered sexual scripts, shaped by sociocultural contexts. Evidence suggests that in conservative contexts, sexual relationships are often structured by traditional gender norms that associate male sexuality with physical pleasure and assertiveness, and female sexuality with emotional intimacy and relational commitment, thereby influencing sexual agency and satisfaction patterns [12,28].

Also, the finding can be explained in light of previous research showing that sexual self-schemas are associated with sexual satisfaction and the way individuals process interpersonal sexual information, including their perceptions of a partner’s sexual satisfaction [29]. This framework may help explain why women in the present study viewed emotional intimacy as an essential component of satisfying sexual experiences and reported dissatisfaction when such intimacy was lacking.

Building on these perspectives, perceptions of “perceived sexual competence”, including attractiveness, sexual skills, and role performance, emerge as one of the key findings, highlighting how idealized sexual scripts are internalized and evaluated within intimate relationships. Within this framework, women’s attractiveness is often disproportionately emphasized as a marker of competence, reflecting entrenched gender biases that may further shape expectations and relational evaluations. Moreover, the gap between internalized schemas and actual marital experiences was found to contribute to reduced satisfaction, echoing prior research that links unrealistic expectations to communication difficulties and diminished sexual well-being [30].

Sexual agency also emerged as a significant factor. Conceptualized as the capacity to recognize one’s needs, communicate desires, and initiate or negotiate sexual interactions [31], agency was predominantly attributed to men, whereas women were more often positioned in passive roles. This asymmetry contributed to sexual dissatisfaction, particularly when women perceived themselves as objects of desire rather than active partners. In some cases, sexual activity was employed strategically, as a means of resolving conflict, sustaining intimacy, or preventing extramarital involvement, thereby underscoring its broader relational functions. These findings suggest that the absence of reciprocal sexual agency may hinder couples’ ability to co-construct mutually satisfying sexual experiences, while simultaneously illustrating the pervasive influence of gendered expectations on intimate dynamics.

One dimension of Sexual agency and control identified in this study was mutual understanding in sex which encompassed activism versus passivity, mutual attunement to sexual emotions, and active interaction in a sexual relationship. Many women reported that sexual activity was expected to be initiated by their husbands, a dynamic that fostered feelings of objectification and, in some cases, sexual dissatisfaction. Consistent with this finding, prior research has demonstrated that women’s experiences of self-objectification in sexual interactions are positively associated with body shame and negatively associated with overall life satisfaction [32]. From a psychological perspective, virginity norms contribute to the formation of culturally informed sexual scripts, which shape the development of sexual schemas and, in turn, influence sexual agency. These processes are embedded within broader sociocultural contexts that often construct gendered expectations, positioning men as more agentic in sexual interactions and women as more passive or sexually restrained. While virginity norms may play a more prominent role in conservative sociocultural contexts such as Iran, the influence of gendered sexual scripts on sexual schemas and sexual agency appears to represent a broader psychosocial process observed across cultures.

As these scripts and schemas are internalized, they may shape how individuals perceive and enact sexual agency in intimate relationships, while sexual agency itself can also influence the interpretation or resistance of these norms in a reciprocal manner. When discrepancies arise between internalized expectations and lived experiences of desire, they may be associated with sexual dissatisfaction, underscoring the complex interplay between virginity norms, sexual schemas, and sexual agency.

Together, these findings demonstrate that sexual satisfaction in conservative societies such as Iran is constrained by entrenched taboos, restrictive sexual scripts, and unequal gender dynamics, and the internalization of these cultural scripts fosters shame and inhibits open communication even within marriage; thus, sexual satisfaction should be understood not simply as an individual psychological outcome, but as a socially mediated phenomenon shaped by norms, discourses, and power relations, underscoring the importance of addressing sociocultural factors in interventions aimed at enhancing sexual well-being.

Building on these findings, several practical implications can be drawn. Sexual education programs in conservative contexts such as Iran should go beyond biological information and explicitly address how cultural taboos, restrictive sexual scripts, and gendered norms shape attitudes toward sexuality. This can help reduce shame and support more open and informed understandings of sexual relationships.

In premarital counseling, attention to these sociocultural influences can help couples recognize how internalized expectations may limit communication and create imbalances in sexual agency, enabling them to develop more realistic and negotiated expectations. Similarly, couple-based interventions can benefit from integrating communication training with discussion of gendered sexual norms, supporting partners in expressing needs, setting boundaries, and reducing misunderstandings rooted in culturally shaped assumptions.

### Limitations

This study has several limitations that should be acknowledged. First, the findings may not be fully applicable to communities with different social and cultural contexts, and further research is needed to examine the transferability of results. The qualitative design also restricts generalizability, underscoring the need for future studies to quantitatively evaluate the relationship between the identified psychosocial factors and newlyweds’ sexual satisfaction before developing health promotion programs.

Second, the recruitment of participants mainly from premarital counseling and healthcare centers may have biased the sample toward health-conscious and educated couples, limiting the representation of those who did not attend such services. The relatively small sample size and reliance on self-reported data, particularly given the sensitivity of sexual topics, may also have introduced bias.

Finally, the dynamics of joint interviews posed challenges. The presence of a female interviewer may have influenced male participants to moderate their disclosures due to cultural taboos. Although individual interviews with male interviewers were offered upon request, some men may still have been cautious in expressing their experiences. Consequently, the apparent early data saturation among male participants may have reflected restricted disclosure rather than comprehensive exploration of their experiences. As a result, men’s perspectives may be underrepresented in the findings, and this limitation should be considered when interpreting the results.

## Conclusion

This study explored the psychosocial determinants of sexual satisfaction among Iranian newlyweds and identified three interrelated categories: premarital sexual norms, sexual schemas, and sexual agency and control. Collectively, these findings suggest that sexual satisfaction is not solely an individual or interpersonal experience but a socially mediated phenomenon shaped by the interaction of cultural norms, internalized sexual beliefs, and individuals’ capacity to exercise sexual agency within intimate relationships. Restrictive social norms and sexual taboos may limit access to sexual knowledge and skills while fostering shame and reinforcing gendered sexual schemas, ultimately influencing the development and expression of sexual agency. These findings highlight the importance of addressing the sociocultural context of sexuality in Iran and suggest that culturally sensitive sexual education, premarital counseling, and couple-based interventions should move beyond providing information alone to also challenge restrictive norms, promote healthy sexual schemas, and strengthen sexual agency to improve sexual satisfaction among newlyweds.

## Supporting information

S1 Table Consolidated criteria for reporting qualitative research: a 32-item checklist for interviews. (DOCX)
