# Supplementary material for: A qualitative exploration of the psychosocial factors of sexual satisfaction in Iranian newlyweds
Source: PLoS One. 2026 Aug 13;21(8):e0356277. doi: 10.1371/journal.pone.0356277 (PMC13472429; doi:10.1371/journal.pone.0356277)
Supplement: Supplementary file 1 [file pone.0356277.s003.pdf]

PLOS One

A qualitative exploration of the psychosocial factors of sexual satisfaction in Iranian newlyweds

--Manuscript Draft--

**Manuscript Number:**	PONE-D-26-20783
**Article Type:**	Research Article
**Full Title:**	A qualitative exploration of the psychosocial factors of sexual satisfaction in Iranian newlyweds
**Short Title:**	Psychosocial factors of sexual satisfaction
**Corresponding Author:**	Ashraf Kazemi<br>Isfahan University of Medical Sciences<br>Isfahan, IRAN, ISLAMIC REPUBLIC OF
**Keywords:**	Psychosocial Factors, Sexual Satisfaction, Newlyweds, Qualitative Research
**Abstract:**	Background: Psychosocial factors related to sexual issues impact sexual satisfaction, and identifying them is beneficial in compiling educational programs for new couples. However, the documents about the psychosocial factors collected in the social context of Iran are inadequate, and the present study was conducted to identify psychosocial factors of sexual satisfaction in newlywed.<br>Method: This qualitative study was conducted with a qualitative content analysis approach on 31 heterosexual newlyweds that less than two years had passed since started having sex. Sampling was implemented using a purposeful method and continued until data saturation. The criterion of data saturation was that no new inferential codes emerged after being repeated at least five times. The data were collected and analyzed through individual and joint semi-structured interviews. The collected data were analyzed using Graneheim and Lundman method.<br>Results: Three main categories were identified: social norms of sexual issues, sexual schemas, and sexual agency. Social norms reflected taboos on sexual self-disclosure, premarital sexual needs, and the obligation of female virginity. Sexual schemas encompassed sexual fantasies, attitudes toward sex, and perceptions of sexual qualification, while sexual agency involved sexual awareness skills, sex as a means of control, and regulation of sexual behavior.<br>Conclusion: The results indicated that a more accurate understanding of the sexual perspectives and ideals of newlyweds and can be utilized in the design of sexual satisfaction programs for newlyweds and helps to compile premarital sex counseling program.
**Order of Authors:**	Zahra Toghiyani
	Ashraf Kazemi
	Mahbobeh Taebi
	Fatemeh Mohammady
**Additional Information:**	
**Question**	**Response**
**Financial Disclosure**<br><br>Enter a financial disclosure statement that describes the sources of funding for the work included in this submission. Review the submission guidelines for detailed requirements. View published research articles from *PLOS ONE* for specific examples.	Yes

This statement is required for submission and **will appear in the published article** if the submission is accepted. Please make sure it is accurate.<br><br>**Funded studies**<br>Enter a statement with the following details:<br>• Initials of the authors who received each award<br>• Grant numbers awarded to each author<br>• The full name of each funder<br>• URL of each funder website<br>• Did the sponsors or funders play any role in the study design, data collection and analysis, decision to publish, or preparation of the manuscript?<br><br>Did you receive funding for this work?	
Please add funding details.<br>as follow-up to "**Financial Disclosure**<br><br>Enter a financial disclosure statement that describes the sources of funding for the work included in this submission. Review the submission guidelines for detailed requirements. View published research articles from *PLOS ONE* for specific examples.<br><br>This statement is required for submission and **will appear in the published article** if the submission is accepted. Please make sure it is accurate.<br><br>**Funded studies**<br>Enter a statement with the following details:<br>• Initials of the authors who received each award<br>• Grant numbers awarded to each author<br>• The full name of each funder<br>• URL of each funder website<br>• Did the sponsors or funders play any role in the study design, data collection and analysis, decision to publish, or preparation of the manuscript?	Isfahan University of Medical Sciences (Grant number: 3401530)

Did you receive funding for this work?"	
Please select the country of your main research funder (please select carefully as in some cases this is used in fee calculation).<br>as follow-up to "**Financial Disclosure**<br><br>Enter a financial disclosure statement that describes the sources of funding for the work included in this submission. Review the submission guidelines for detailed requirements. View published research articles from *PLOS ONE* for specific examples.<br><br>This statement is required for submission and **will appear in the published article** if the submission is accepted. Please make sure it is accurate.<br><br>**Funded studies**<br>Enter a statement with the following details:<br>• Initials of the authors who received each award<br>• Grant numbers awarded to each author<br>• The full name of each funder<br>• URL of each funder website<br>• Did the sponsors or funders play any role in the study design, data collection and analysis, decision to publish, or preparation of the manuscript?<br><br>Did you receive funding for this work?"	IRAN, ISLAMIC REPUBLIC OF - IR
**Competing Interests**<br><br>Use the instructions below to enter a competing interest statement for this submission. On behalf of all authors, disclose any competing interests that could be perceived to bias this work—acknowledging all financial support and any other relevant financial or non-financial competing interests.<br><br>This statement is required for submission and **will appear in the published article** if	The authors declare that they have no conflict of interest.

the submission is accepted. Please make sure it is accurate and that any funding sources listed in your Funding Information later in the submission form are also declared in your Financial Disclosure statement.<br><br>View published research articles from *PLOS ONE* for specific examples.<br><br>**NO authors have competing interests**<br><br>Enter: *The authors have declared that no competing interests exist.*<br><br>**Authors with competing interests**<br><br>Enter competing interest details beginning with this statement:<br><br>*I have read the journal's policy and the authors of this manuscript have the following competing interests: [insert competing interests here]*<br><br>* typeset	
**Ethics Statement**<br><br>Enter an ethics statement for this submission. This statement is required if the study involved:<br><br>• Human participants<br>• Human specimens or tissue<br>• Vertebrate animals or cephalopods<br>• Vertebrate embryos or tissues<br>• Field research<br><br>Write "N/A" if the submission does not require an ethics statement.<br><br>General guidance is provided below. Consult the submission guidelines for detailed instructions. **Make sure that all information entered here is included in the Methods section of the manuscript.**	Ethics Committee of Isfahan University of Medical Sciences<br>IR.MUI.NUREMA.REC.1401.130<br>rmed consent was obtained from all participants

**Format for specific study types**<br><br>**Human Subject Research (involving human participants and/or tissue)**<br>• Give the name of the institutional review board or ethics committee that approved the study<br>• Include the approval number and/or a statement indicating approval of this research<br>• Indicate the form of consent obtained (written/oral) or the reason that consent was not obtained (e.g. the data were analyzed anonymously)<br><br>**Animal Research (involving vertebrate animals, embryos or tissues)**<br>• Provide the name of the Institutional Animal Care and Use Committee (IACUC) or other relevant ethics board that reviewed the study protocol, and indicate whether they approved this research or granted a formal waiver of ethical approval<br>• Include an approval number if one was obtained<br>• If the study involved *non-human primates*, add *additional details* about animal welfare and steps taken to ameliorate suffering<br>• If anesthesia, euthanasia, or any kind of animal sacrifice is part of the study, include briefly which substances and/or methods were applied<br><br>**Field Research**<br><br>Include the following details if this study involves the collection of plant, animal, or other materials from a natural setting:<br>• Field permit number<br>• Name of the institution or relevant body that granted permission	
Data Availability<br><br>Before publication, Authors are required to make fully available and without restriction all data underlying their findings. Sharing the data underlying your findings increases the visibility, credibility, and impact of your work, while supporting transparency and future scientific discovery. Please see our PLOS Data Policy page for detailed information on this policy.</p><br>A Data Availability	o data was generated by this study. But because of the ethical code data will be available on request from the corresponding author, Ashraf Kazemi, E-mail: kazemi2nm.mui.ac.ir<br>Isfahan University of Medical Sciences

Statement, detailing where the data can be accessed, is required at first submission. Insert your Data Availability Statement in the box below.</p><br><br>Please see the data reporting of our submission guidelines for instructions on what you need to include in your Data Availability Statement. Examples of acceptable Data Availability Statements are included here for reference:</p><br> <br> - The minimal data set [and accompanying code, where generated] is available at [repository name] via [DOI hyperlink] <br> - No data was generated by this study. The following existing data sources were used: [dataset name] from [dataset location] available via [web address or DOI hyperlink]. <br> - For Study Protocols: No datasets were generated or analysed during the current study. All relevant data from this study will be made available upon study completion. <br> <br><br><i>PLOS allows rare exemptions to address legal and ethical concerns. If you have legal or ethical restrictions, please use the box below to detail these in full sentences for the Journal team to consider.</i></p><br><br>This statement will be typeset if the manuscript is accepted for publication.</p>	

Manuscript Click here to access/download;Manuscript;manuscript (marked).docx

Title: A qualitative exploration of the psychosocial factors of sexual satisfaction in Iranian newlyweds

Running title: Psychosocial factors of sexual satisfaction

Authors

Zahra Toghiyani[1], Ashraf Kazemi[2], Mahbobeh Taebi[2], Fatemeh Mohammady[2]

1: Student research committee, School of Nursing & Midwifery, Isfahan University of Medical Sciences, Isfahan, Iran. E-mail: z.toghiyani@nm.mui.ac.ir

2: Nursing and Midwifery Care Research Center, Isfahan University of Medical Sciences, Isfahan, Iran. E-mail: kazemi@nm.mui.ac.ir

Corresponding author: Ashraf Kazemi,

Addressee: Reproductive Health Department, School of Nursing and Midwifery, Isfahan University of Medical Sciences, Hezarjerib AV. Isfahan, Iran.

Telephone number: 0098-31-37927537

Fax number: 0098-311- 668 7898

E-mail: kazemi@nm.mui.ac.ir

Telephone number: 0098-31-37927537
Fax number: 0098-311- 668 7898

Conflict of interest

The authors declare that they have no conflict of interest.

Abstract

Background: Psychosocial factors related to sexual issues impact sexual satisfaction, and identifying them is beneficial in compiling educational programs for new couples. However, the documents about the psychosocial factors collected in the social context of Iran are inadequate, and the present study was conducted to identify psychosocial factors of sexual satisfaction in newlywed.

Method: This qualitative study was conducted with a qualitative content analysis approach on 31 heterosexual newlyweds that less than two years had passed since started having sex. Sampling was implemented using a purposeful method and continued until data saturation. The criterion of data saturation was that no new inferential codes emerged after being repeated at least five times. The data were collected and analyzed through individual and joint semi-structured interviews. The collected data were analyzed using Graneheim and Lundman method.

Results: Three main categories were identified: social norms of sexual issues, sexual schemas, *and* sexual agency. Social norms reflected taboos on sexual self-disclosure, premarital sexual needs, and the obligation of female virginity. Sexual schemas encompassed sexual fantasies, attitudes toward sex, and perceptions of sexual qualification, while sexual agency involved sexual awareness skills, sex as a means of control, and regulation of sexual behavior.

Conclusion: The results indicated that a more accurate understanding of the sexual perspectives and ideals of newlyweds and can be utilized in the design of sexual satisfaction programs for newlyweds and helps to compile premarital sex counseling program.

Keywords: Psychosocial Factors, Sexual Satisfaction, Newlyweds, Qualitative Research

In introduction section, you should discuss the specific perspective on these issues. For example, quality of marital life, emotional divorce, or a consultation program for newlyweds. Social and cultural factors would affect sexual behaviors. All of the epidemiologic studies showed and stressed the social factors on health behaviors. These are clear in past studies and the majority of epidemiologic studies. I suggest " consultation programming" before and after marriage, you choose. It would be an important perspective and applicable for healthcare providers.

Introduction

Sexual satisfaction is a significant component of sexual health, which increases the quality of couples' lives by creating marital satisfaction [1] and helps reinforce marital life [2]. However, the lack of sexual satisfaction threatens society's health by generating a complex network of social, physical, and psychological complications.

Decreased marital satisfaction can increase the probability of depression [3], and decreased self-esteem [4], and sexual dysfunction. The increase in the likelihood of social difficulties, such as sexual extramarital relations [5], and the instability of married life, have altered this issue to a social health problem. Moreover, sexual dissatisfaction is associated with an increased emotional divorce [6]. In this situation, although couples live together and perform their marital duties, they are separated from each other emotionally, psychologically, and spiritually [7].

The adverse and significant effects of sexual dissatisfaction have drawn researchers' attention to identifying its determining factors. A Study in this field has shown that in the first years of marriage, couples' experiences of sexual relations are an essential factor in marital satisfaction and maintaining married life This finding shows that newlyweds can be a proper target population for identifying factors related to sexual satisfaction [8]. Therefore, it is necessary to examine the factors related to sexual satisfaction shortly after the beginning of sexual intercourse.

Although the cycle of sexual response, followed by sexual pleasure, goes through a physiological process during intercourse, the impact of environmental factors on sexual satisfaction has converted it into a bio-psycho-social issue [9], and identifying its psychosocial factors in the first years of marriage provides richer information.

In Iran, cultural and social discourses, reinforced through family, education, and policy, contribute to a social reality in which sexuality is largely privatized and moralized. Empirical studies confirm that taboos manifest in parents' conservative approaches to sexuality education for their children

[10], and in reluctance or outright inability of engaged or newly married couples to receive or seek out accurate information or express mutual sexual needs. For instance, a qualitative study of engaged couples in Iran showed that socio-cultural constraints and inadequate knowledge are key challenges [11].

The difference in societies' sociocultural backgrounds accounts for the need for a deep understanding of psychosocial factors affecting sexual satisfaction. Compared to modern countries, in traditional societies such as Iran, the development of sexual concepts as a cognitive generation about sexual aspect, and women's and men's attitudes has been formed in a sociocultural context, which has a dissimilar view on sexual issues.

In such societies, it is necessary to have an in-depth identification of the attitudes related to sexual satisfaction in newlyweds based on their experiences. In the Iranian context, where cultural norms, social values, and family structures significantly shape marital dynamics, understanding the psychosocial determinants of sexual satisfaction is particularly critical [12]. Despite the importance of this issue, there remains a scarcity of empirical research exploring the intersection of psychosocial factors and sexual satisfaction within Iranian society.

Really? Are you sure it can be conducted as an empirical stu dy on these factors? it is not suitable for your study, and here .

Conducting research in this area is essential not only for advancing scientific knowledge but also for providing culturally sensitive insights that can inform counseling, educational programs, and interventions aimed at strengthening marital relationships. By identifying the key psychosocial predictors of sexual satisfaction among newlywed Iranian couples, researchers and practitioners can contribute to promoting healthier marriages, reducing marital conflicts, and enhancing the overall quality of family life.

Since a qualitative study allows one to examine and interpret how individuals interact with different subjects in their specific social context [13], this approach is appropriate for deeply understanding the psychosocial factors related to sexual satisfaction. Accordingly, the present study aims to identify the psychosocial determinants of sexual satisfaction among Iranian newlyweds, focusing specifically on couples who have been sexually active for less than two years. This early stage of a relationship is a dynamic period during which sexual and psychosocial

patterns are still under formation and are more susceptible to fluctuation. Sexual satisfaction in this period is especially sensitive to interpersonal expectations, emotional intimacy, and prevailing cultural norms, and may change rapidly before becoming relatively stable [14]. By concentrating on couples in this formative phase, the study seeks to capture the initial psychosocial contributors to sexual satisfaction without the confounding influences of long-term marital adjustments, parenthood, or the gradual habituation that can accompany enduring relationships.

your study is qualitative, so you can't judge the "effect" of those factors on satisfaction.

The present study sought to address the following question: How do psychosocial factors, including interpersonal and social communication, social norms, and expectations, affect sexual satisfaction among newly married Iranian couples.

Methods and Materials

This qualitative study was conducted in Isfahan Iran, with the approval of the ethics committee of Isfahan University of Medical Sciences (IR.MUI.NUREMA.REC.1401.130). All methods were performed in accordance with the relevant guidelines and regulations.

An inductive qualitative approach was chosen to explore psychosocial factors of marital sexual satisfaction because it allows themes and concepts to emerge directly from couples' lived experiences, without being constrained by pre-existing theories. Given the sensitive and context-dependent nature of sexual satisfaction, this approach increases the likelihood of uncovering novel, culturally specific, and nuanced psychosocial variables. It supports developing a rich, authentic understanding grounded in the data, enhancing credibility and relevance of findings in the study's specific social context [15].

Researcher Characteristics and Reflexivity

The research team comprised experts with professional backgrounds in qualitative research, reproductive health, psychology, and sexual health. Both male and female researchers were involved, bringing diverse perspectives that enriched the interpretive process. Their prior assumptions were grounded in professional experience and theoretical knowledge of intimate

relationships and sexual well-being, but they remained attentive to minimizing potential bias through reflexive practice. Importantly, the researchers conducted their professional activities independently from any institutions or service centers directly related to the study setting. This independence helped reduce potential conflicts of interest and enhanced the credibility and trustworthiness of the research process.

Participants

The participants in this study included 31 heterosexual Iranian women and men who had been sexually active with their spouses for less than two years. Participants were recruited among clients attending premarital education centers, health service centers, and sexual counseling centers. In Iran, all couples are required to participate in premarital counseling before marriage, and after marriage they have a health record in a city health center. Therefore, these centers offer access to the target population and enable sampling with maximum diversity.

The inclusion criteria included having no known mental and behavioral disorders or chronic physical diseases affecting sexual relations. Also, because of the effect of the pregnancy and infertility on the sexual satisfaction, these criteria were the exclusion criteria. As some couples in Iran may delay cohabitation and the initiation of sexual activity following official marriage registration, individuals whose marriages had been formally registered for more than five years were excluded from the study.

Sampling

Here, draw the characteristics of participants (sex, age, etc.) in a table.

The sample size for this qualitative study was determined according to the principle of data saturation. Data collection continued until no new inferential codes emerged and until the existing inferential codes recurred in at least five separate interviews. To enhance the richness and credibility of findings, participants were purposefully selected to ensure maximum diversity in terms of age, educational level, and duration since the initiation of sexual activity. This approach allowed the study to capture a broad spectrum of experiences while providing sufficient depth to achieve thematic saturation, which served as the primary criterion for concluding data collection.

Procedure and Data collection

This section would be summarized. You should omit the repeated sentences, and you should stress the important points that are necessary for this section.

Participants were identified via the intake units at premarital counseling centers and health service centers. After introducing the researcher and explaining the study's purpose, eligibility criteria were evaluated. For those who met the inclusion criteria, the research process, ethical considerations, and their rights as participants were explained. The interviewer was mindful that discussing personal sexual experiences could cause stress or discomfort for participants due to the sensitivity of the topic. To address this, interviews were conducted in a private and respectful environment, and participants were reminded of their right to skip any questions or withdraw at any time without consequence. In addition, clear assurances of confidentiality and anonymity were provided, and the researcher adopted a supportive, non-judgmental approach to foster trust and minimize potential distress.

Also, for joint interviews, both partners provided written consent. The timing and location of each interview were arranged by mutual agreement between researcher and participant. All interviews took place in a private room at the centers. Interview scheduling was based on the participants' preferences. During the interview sessions, only the interviewer and the participant, or couple, in the case of joint interviews, were present. Due to the desire of two male participants, two interviews were conducted by a male interviewer with a master's degree in psychology.

In this study, data were collected through physical face-to-face, semi-structured, in-depth interviews conducted either individually or in dyadic settings. The semi-structured format allowed for a balance between consistency across interviews and flexibility to pursue participants' unique perspectives and experiences in greater depth. Open-ended guiding questions were developed to ensure coverage of the main research objectives, while allowing participants the freedom to elaborate and introduce new themes. Individual interviews provided a private space for participants to share personal insights, whereas dyadic interviews created opportunities to explore shared experiences, interactions, and relational dynamics between participants. All interviews were conducted in a conversational manner to foster trust and encourage rich, nuanced accounts.

In total, 31 individual interviews were conducted with 19 women and 12 men, followed by 5 couple interviews involving 10 participants who had previously taken part in the individual interviews. Guiding questions were used in interviews. To prepare the guide questions, an in-depth, unstructured interview was conducted with five eligible individuals and the interview guide was validated by two experts. Data from these interviews were not included in the data analysis. Before these interviews, the researchers conducted a comprehensive study on the psychosocial factors of sexual satisfaction, and the data were used to guide the interviews and better understand the data. The interviews were done by one of the researchers.

The questions, such as "Please tell us about the experiences that shaped your sexual thoughts" and "Tell us about your experiences with sex" were developed based on three unstructured interviews, whose data were not used in the study's data analysis.

Based on these interviews, a set of guiding questions was developed. Examples include:

- In your social interactions with family members and others, what kinds of understandings about sexuality have been formed for you?
- If you wished to discuss sexual matters with your close relatives, what kinds of behaviors would you expect to observe?
- When you experience sexual needs or desires, how do you usually respond or manage them?
- In what ways did your family, cultural background, or upbringing shape your attitudes toward sexuality and sexual satisfaction?
- How do societal or community norms influence your sexual attitudes, behaviors, and satisfaction?

Moreover, probing questions, such as "Tell us about your feelings at the time" and "What was your impression" were used to deepen the interviews. The interviewer also documented non-verbal expressions, including body movements and gestures that conveyed participants' emotions during the interviews, as part of the qualitative data collection process. In order to prevent the bias of the spouse's presence in couple interviews, an attempt was made to deepen the interviews with questions such as "What do you think of your spouse's statements?"

Joint interviews were conducted in a private and comfortable setting to promote openness and reduce potential discomfort. Each session began with general, non-sensitive questions to establish rapport and gradually progressed to more specific inquiries regarding psychosocial factors influencing sexual satisfaction, including communication patterns, emotional intimacy, cultural or religious values, body image, and stress. Examples of the questions include:

Interviews were semi-structured, guided by open-ended questions that allowed participants to elaborate freely while encouraging dialogue between partners.

- Can you tell me a little about your relationship history and how long you have been together?
- How do you usually talk about your sexual needs and desires with each other?
- Are there moments when you and your partner view sexual satisfaction differently? Can you give an example?
- Do you feel there are topics we haven't asked about that are important to understanding your experiences as a couple?

The interviewer maintained neutrality, ensured balanced participation, and attended to both verbal and non-verbal interactions within the dyad.

Confidentiality of participants was strictly maintained throughout the study. Each participant was assigned a pseudonym, and all identifying information was omitted from transcripts and reports. Interviews were conducted in private settings, with only the interviewer and participant (or participating couple) present. Audio recordings were accessible solely to members of the research team, stored in password-protected digital files, and other data (notes, transcripts) were secured similarly; no unauthorized person had access to these recordings. These confidentiality procedures were thoroughly explained to participants during the informed consent process, in accordance with ethical guidelines.

Also, it was explained to the participants that the responsibility for maintaining the confidentiality of their information rests with the principal investigator. The right to refuse to

participate or withdraw from the study at any time, and no impact of their decision to accept or reject the researchers' invitation on the center's routine care were explained to the invitees. It was also clarified that they would have the right to choose the interviewer's gender, and the results of the study would be presented anonymously. Also, if certain circumstances arose where the participants wanted to stop the interview, the interview was stopped and postponed to another time. In joint interviews, if one partner expressed a wish to discontinue the joint session, the interview was immediately stopped. If the other partner still wished to proceed, the interview was resumed individually. The individual interviews lasted between 50 and 70 minutes, while the couple interviews ranged from 60 to 85 minutes in duration. Sampling started in 23-July 2023 and continued until 18- January 2024.

Data analysis

In this section, you should, based on Grranheim & Lundman's method, present the steps of analysis. Also, you would be designing the table with quotations, codes, and categories. In other words, readers could understand how you do the steps of analysis.

Manual analysis of the data was carried out using Graneheim and Lundman's method [16] in parallel with sampling. Shortly after each interview, the audio file was listened to several times and was transcribed verbatim by the first author. Previously recorded observations were noted next to the narrations. The first and second authors read the transcribed text and field notes several times to ensure a thorough understanding of the content.

Meaning units were identified as words, sentences, or paragraphs that related to the same central meaning. Each meaning unit was condensed, i.e., shortened while preserving the core meaning. This step allowed us to reduce redundancy without losing essential content. For example, a woman stated that "Breaking the hymen before marriage, for whatever reason, is a disaster. Since childhood, my mother always reminded me to be careful not to harm my hymen while playing. If my hymen were broken (when I was single), everyone would think that I had a relationship with someone." This was coded as "the stigma of breaking the hymen before marriage.

Codes were compared for similarities and differences. Related codes were grouped into sub-categories, and then broader categories, which represented the manifest content of the data. Categories were further interpreted to formulate themes, representing the latent meaning of the text.

The first round of analysis generated 720 codes across all transcripts. This large set reflected the richness of the data and ensured that no nuance was missed. Codes were reviewed for redundancy, overlap, and conceptual similarity. Similar codes were merged. Through constant comparison, 720 codes were condensed into 234 unique and meaningful codes. The 234 codes were clustered into sub-categories (e.g. Obligation to preserve virginity before marriage). These sub-categories were then abstracted into three overarching categories.

Interview transcripts of the joint interviews were analyzed using thematic analysis with individual-level coding. Each partner's responses were first coded separately to capture unique perspectives and experiences. Subsequently, a comparative process was undertaken to examine convergence and divergence between partners' accounts. This two-step approach allowed for the identification of both individual meanings and relational dynamics, highlighting how sexual satisfaction is perceived, negotiated, and co-constructed within the couple. Analytical memos were used to document observations of interactional patterns and contextual factors emerging from the dyadic setting, thereby ensuring a comprehensive interpretation of the psychosocial influences on sexual satisfaction.

Trustworthiness

In order to ensure the accuracy and rigor of the data, four criteria of credibility, dependability, transferability, and confirmability were used. [17]. To enhance the credibility of the findings, an audit trail of the reduction process was maintained in a coding matrix, documenting how meaning units, condensed text, codes, and categories were linked. Research team members independently coded portions of the data and then met to discuss discrepancies until consensus was achieved. Peer debriefing and iterative discussions ensured the stability and trustworthiness of the final categorization.

Also, encompassed external peer review, long-term engagement with the participants, and participant review strategies were implemented. To ensure the adaptability of the data, the experts' opinions were used. Also, the dependability of the data was strengthened through a comprehensive description of each research stage. To enhance the transferability of the data, the research findings were made available to three new couples for assessment regarding the alignment between the findings and their own experiences. To enhance the confirmability of the data, three interviews were reviewed by four external experts in qualitative research, two specializing in reproductive health and two in psychology. These experts were tasked with verifying the accuracy of the coding process.

Results

Of the 34 eligible individuals invited for the study, 31 individuals including 14 women and 7 men participated in Individual interviews and 5 couples participated in joint interviews .The participants included 19 women and 12 men with the mean age of 25.1 and 30.2 years respectively. The characteristics of the participants are presented in Table 1. Set it in the text, in the participant section.

The analysis of the participants' experiences led to the extraction of 720 inferential codes. After merging similar codes, the number of codes was reduced to 234. The extraction of three main categories of "Social norms of sexual issues," "Sexual schemas," and "Sexual agency" was the result of analyzing the data in the study population (Table 2).

1. Social norms of sexual issues

This category was derived from three subcategories: 'the taboo of sexual self-disclosure before marriage,' 'social norms of meeting sexual needs before marriage,' and 'obligation to preserve virginity in women before marriage.'

1.1. ***The taboo of sexual self-disclosure before marriage***

The main cods, 'the taboo of sexual self-disclosure before marriage,' was inferred from three main cods: 'embarrassment about obtaining sexual information,' 'perceiving the sinfulness of sexual needs,' and 'embarrassment about sexual demands.' These main cods were extracted from

participants' statements and indicated the taboo of social issues in society that caused the participants to consider sexual issues before marriage as immoral and feel embarrassed to express their need for sexual information.

Moreover, the taboo of expressing sexual issues made some participants, especially during adolescence, feel guilty about their sexual emotions and embarrassed about expressing their need to satisfy their sexual desires. One of the men (P2) stated: "*At the beginning of my married life, I felt there was a lot that I needed to know about sexual issues. But in our family, we weren't allowed to discuss such issues. Once my uncle said something about AIDS, my father got up and said angrily: "Shame on you. You shouldn't talk about these things in public; family is respected. I didn't even think about asking my Dad about sex."*

1.2. ***Social norms of meeting sexual needs before marriage***

The category of social norms of premarital sex was derived from the three main cods of 'sinfulness of sexual thoughts,' 'sinfulness of masturbation,' and 'the value of sexual restraint.'

The sinfulness of sexual thoughts was inferred from the statements of the participants who said that they felt guilty by thinking about sexual topics and remembering the sexual scenes that they had seen willingly or unwillingly and tried to get rid of those thoughts. They stated that sometimes they would satisfy their sexual needs by having sexual thoughts in their privacy or by masturbating; however, they were ashamed and felt guilty about it. In this regard, the woman (P15) stated: *"When I was obsessed with sexual thoughts, it was then beyond my control. I felt that the devil was roaming around and pushing me through these thoughts; I felt helpless. After that, I asked God to forgive me."*

The value of sexual restraint was also extracted from statements showing that individuals were in a social context where sexual abstinence and suppression of sexual needs were considered social

value. This social value was formed by establishing social messages that reflected the standard of perfection in women and men as overlooking sexual needs. In this regard, a man (P16) stated: *"I was involved (in sexual excitement) before marriage. …., but I couldn't help it. "I had to suppress it; otherwise, it would mislead me. If I gave in, I'd lose control and be unable to resist. I'd end up ensnared by it, whether through masturbation or other shameful acts."*

1.3. ***Obligation to preserve virginity before marriage***

Obligation to preserve virginity was one of the subcategories from which the category of social norms of sexual issues was extracted. This sub category was derived from the main cods of 'the stigma of breaking the hymen before marriage,' 'the requirement of confirming virginity in sexual relations after marriage,' and 'the feeling of being sexually threatened by men.'

The perception of the stigma of breaking the hymen before marriage was extracted from experiences showing that the female participants received messages since their childhood that breaking the hymen was associated with negative social judgments about girls and their families. Therefore, preventing it from breaking before marriage was considered a social duty for girls and family members, and breaking the hymen during the first intercourse with the husband indicated the chastity of the woman. The woman (P9) expressed her experiences in this regard as follows: *"Breaking the hymen before marriage, for whatever reason, is a disaster. Since childhood, my mother always reminded me to be careful not to harm my hymen while playing. If my hymen were broken (when I was single), everyone would think that I had a relationship with someone or that I was raped. I wasn't considered a virgin."*

The analysis of the interviews showed that the social value of virginity and the commitment to preserve virginity had caused men to be perceived as potential aggressors, and in order to foil this threat, women were obliged to avoid the possibility of harm in regular social relationships and

interactions. Based on these experiences, ‘Feeling of being sexually threatened by men’ was deduced. For instance, a woman (P8) said: *“Since I was a child, the family didn’t allow me to go alone where there were men or boys, even to relatives’ houses. One day, I was playing outside the house with a boy. My brother came and saw me. He took my hand and took me home. He said: “Didn’t I tell you not to play with boys?” He also told my mom not to let me play outside anymore. A mishap (sexual rape) would happen to me, which would cause disgrace. Because of that, I was always afraid of men.”*

2. Sexual schemas

Sexual schemas were inferred from the subcategories: ‘sexual fantasies,’ ‘attitudes towards sex,’ and ‘sexual qualification.’

2.1. Sexual fantasies

Sexual fantasies were extracted from the main cods: ‘sexual diversification,’ ‘sexual fantasizing,’ and ‘seeking affection versus pleasure.’

Sexual diversification was extracted from narratives indicating that a pleasurable sexual relationship was not necessarily defined as penile-vaginal intercourse. Some participants recognized the desire for sex in a variety of ways as a measure of pleasurable sex and molded their sexual dreams accordingly.

One of the man participants (P6) stated, “*Before marriage, I imagined sex differently, just like in erotic movies. I would have sex in diverse ways in my sexual imagination. I even thought that the orgasm would be much more enjoyable than this*.” When asked how this perception of sex has affected your sex life, this participant replied, “*Our sex is not what I thought it would be; repeated sex; I’m satisfied, and that’s it*.”

In this regard, a woman (P14) stated that *"My husband says that he doesn't enjoy sex with me anymore. It's boring. He expects me to do the dirty things that the pornographic actresses do."*

2.2. Attitude toward sexual issues

Attitudes toward sexual issues were inferred from: 'Attitudes toward sex,' 'Sexual expectations,' and 'Sexual obedience'. 'Attitudes towards sex' was concluded from narratives that reflected individuals' feeling about sexual issues. One of the women (P3) expressed her experiences: *"I have a traditional family, and we can't talk about these issues in our company. Once, I said to my mother what the schoolchildren had said about male-female sexual relations. My mother said: "These are bad words. I don't want to hear you talk about them again."*

The main code 'Expectancies' refers to the prospects the participant expected from the sexual relationship. Some participants considered the outcome of sex an essential and valuable element, and others did not value it. The sexual expectations refers to the participants' expectations of ideal sex. Some expected to have penile-vaginal intercourse, and some considered reaching orgasm in sexual intercourse as the main goal of sexual intercourse, and they expected their spouse to care about this issue during sexual intercourse. In this regard, a woman (P1) said: *"Sexual relationship is a part of marital life. I don't care how it passes.*

Sexual obedience was another main code that shaped the attitude toward sexual issues. This subcategory mentioned the gender attitude that the participants had toward their and their spouse's sexual duties. Some believed that women had the duty to satisfy their husband's sexual desires. Some others believed that both of them were required to respect each other's sexual desires during sex and make an attempt to fulfill them. In this regard, a woman participant (P11) stated: *"In our married life, sometimes I don't want to have sex. But I do it because every woman is obliged to*

(sexually) satisfy her husband. I don't feel good about this (sexual) relationship, but I am satisfied because I am fulfilling my duty."

2.3. Sexual qualification

The subcategory of 'sexual qualification' was extracted from the main cods of 'sexual attractiveness,' 'skills of sexual behavior,' and 'playing sexual roles.'

These main cods were extracted from narratives indicating that in order to have an ideal sexual relationship, the adaptation of behaviors and the attractiveness of the sexual organs and even the shape of the body with the social criteria of attractiveness, as well as the requirement to have the skill of sexual behaviors in some cases, were required.

One of the woman participants (P8), who considered her lack of sexual attractiveness as the reason for her husband's low sexual satisfaction, said: *"Who can be excited by this thin body with small breasts? My husband likes my breasts to be uplifted. Maybe in that case, my husband would like to have more sex."*

Another participant (P20) said, "*My husband doesn't know how to satisfy me. When I object to him, he expects me to try it myself. He must know how to have intercourse so that I can be satisfied.*" This participant continued, "*How would I know that? Men easily talk about sexual issues and learn from each other. But learning these things (sex) is against the custom for women, especially before marriage*."

3. Sexual agency

Sexual agency was another category extracted from the data and the three subcategories of 'sexual knowledge and skills,' 'sex as a means of control,' and 'controlling sex.'

3.1. Sexual awareness and skills

The subcategory of sexual awareness and skill was derived from the main cods of 'Sexual awareness,' 'the skill of expressing sexual needs and demands,' and 'the skill of satisfying sexual

needs.' Regarding the sexual awareness, some participants stated that they lacked the necessary knowledge to be in sexual relations due to social and cultural barriers before marriage. In order to have an ideal sex life, they felt it necessary to gain more knowledge about their sexual issues. In this regard, a man participant (P25) said: *"My wife complains that she doesn't have an orgasm in sex. She believes I don't know how to satisfy her. I tell her that she herself should know what to do to have an orgasm. Everyone should learn how to enjoy their relationship (sexual relationship)."*

In addition to sexual awareness, the skill of expressing sexual needs and demands was another main code of sexual awareness and skills subset. This code was specifically extracted from the analysis of sexual experiences of women who stated that due to the taboo of sexual issues, they had not been able to acquire skills in expressing their needs and demands and believed that their unexpressed sexual needs should be met by their spouse. However, men mainly believed their wives needed to express their needs and demands. In this regard, a woman participant (P26) stated: *"Many times, I desire to have sex, but I can't tell my husband directly because I'm embarrassed. I don't even know how to show that I need it with my behavior. When I complain about it, he says that he's not a mind reader and that I should somehow say or at least show it. But I think he should figure it out."*

The skill of satisfying sexual needs was another subcategory that formed the main cods of sexual awareness and skills. The analysis of the participants' experiences showed that some individuals believed that in order to have pleasurable sex, both partners needed to have the skills to meet their sexual needs and be actively involved in this relationship. Some others believed that the sexual partner needed to have enough skills to meet their spouse's sexual needs. In this regard, a woman (P22) stated: *"My husband says that I should know how to do it myself to be able to achieve sexual pleasure. He says that he doesn't have any problems, and he knows how to satisfy himself."*

3.2. Sex as a means of control

The subcategory of 'Sex as a means of control' was extracted from the main cods of 'Means of resolving marital conflicts,' 'prevention of extramarital relations,' 'maintaining marital intimacy,' and 'sexual authoritarianism: a means of confirming authority.'

The main cods of 'Means of resolving marital conflicts' was extracted from experiences indicating that some sexual partners had sexual intercourse to establish a new emotional relationship or to appease the spouse after marital conflicts or disputes. In this regard, a woman participant (P23) who believed her husband lacked proper understanding of the cause of the conflict said: *"When we have an argument, my husband has sex that night. He believes that everything will be solved this way. "Whenever he wants our relationship to feel intimate, he initiates sex, so that he doesn't have to respond to many other issues."*

Another main code that shaped 'Sex as a means of control' was prevention of extramarital relations. This subcategory was extracted from the inferential codes showing that some women considered sex with their spouse as a means to prevent extramarital affairs by their husband. In this regard, a woman (P19) said: *"Men have higher (sexual) needs than women. He will go out with someone else if I don't meet them. I have to accept sex to preserve my married life, even if I don't like it or I'm ready for it."*

By analyzing the experiences of some participants, the main code 'maintaining marital intimacy' was extracted, which showed that for some individuals, sex was a way to achieve and maintain intimacy with their spouse. In this regard, a woman (P3) said: *"When we have sex, I feel that I love him more, and he loves me more, and I can trust him more. This shows that he still loves me."*

The subcategory sexual authoritarianism; a means of confirming authority' refers to the experiences of participants who expressed that despite their reluctance to have sex, their husbands

tried to show their authority in their lives by having sex. In this regard, a woman (P1) said: *"Sexual intercourse that is meant to show dominance is not satisfactory. Despite my unwillingness, my husband has sex with me to show that what he says goes."*

3.3. Controlling sex

Another subcategory shaping sexual agency was 'Controlling sex,' which was extracted from the main cods of 'Activism versus passivity', 'Mutual understanding of sexual emotions,' and 'Active interaction in a sexual relationship.'

The main cods main cod of 'activism versus passivity' was derived from the statements that couples made regarding their sexual relationship. The analysis of the data showed that men actively participated in sex, while women had sex passively in response to men's sexual behavior. In this regard, a man participant (P16) stated: *"I always do something (sexual behaviors); she waits for me to do it to make her satisfied. In sex, women are more passive, and men are active. Sex is a masculine skill."*

The 'Mutual understanding of sexual emotions was derived from the statements of the participants indicating that, in a sexually satisfying relationship, they needed to understand their spouse's sexual reactions. The main cod of 'active interaction in a sexual relationship' was inferred from the participants' statements, indicating that mutual active participation was a significant factor in a satisfactory sexual relationship. For instance, a man (P18) stated: *"My wife shows no reaction during sex. I like her to show it if my stimulations cause her sexual pleasure. This way, I will be more excited and have more sexual pleasure. She must do something during sex."*

Discussion

This section should be based on the " problem statement".Because of my opinion in that section, I suggest regulating these findings. Your discussion is based on evidence that was known. So, I stress my idea in the first section that I mentioned above.

The findings of this study highlight the central role of psychosocial factors, particularly social norms, sexual schemas, and sexual agency, in shaping sexual satisfaction among Iranian

newlyweds. Consistent with social constructionist perspectives, sexual experiences were not merely individual or biological but socially constructed through dominant cultural narratives, religious traditions, and gendered expectations [18].

A key theme was the persistence of social taboos surrounding sexuality. From childhood through adulthood, participants internalized prohibitions against premarital sexual expression, resulting in feelings of guilt, shame, and anxiety regarding sexual thoughts, desires, and behaviors. This taboo was strongly associated with restricted communication, lack of reliable sexual knowledge, and unrealistic marital expectations, thereby reducing opportunities for intimacy and satisfaction.

The cultural emphasis on female virginity prior to marriage further reinforced restrictive norms, framing women's sexual purity as a symbol of family honor and positioning men as potential threats to this purity. These findings align with sexual script theory, which posits that societal narratives dictate acceptable sexual roles: men are granted sexual agency, while women are expected to embody modesty, passivity, and compliance [19]. Consistent with the results of the present study, other studies reported that in some societies, the taboo of sexual issues has limited the acquisition of information from reliable sources [20-22].

"Another finding of the qualitative study was the concept of sexual schema. Sexual schema is a concept with a multidimensional structure that provides a holistic theoretical perception of sexual aspects; that organize individuals' beliefs, expectations, and experiences related to sexuality, were also identified in this study [23]. In particular, this concept was derived from the subcategories of sexual fantasies, attitudes toward sexual issues, and sexual qualification. Within this context, gender differences in sexual schemas were evident. Women prioritized emotional intimacy in their sexual experiences and reported dissatisfaction when such intimacy was absent, whereas men were

more oriented toward physical pleasure and variety. These mismatches in expectations illustrate the influence of gendered sexual schemas on marital satisfaction.

Moreover, perceptions of “sexual qualification”, encompassing attractiveness, skills, and role performance, emerged as a key determinant of satisfaction. Notably, women’s attractiveness was disproportionately emphasized as a marker of competence, reflecting entrenched gender biases. The gap between internalized schemas and actual marital experiences was found to contribute to reduced satisfaction, echoing previous research linking unrealistic expectations with communication difficulties and diminished sexual well-being.

Sexual agency also emerged as a significant factor. Conceptualized as the capacity to recognize one’s needs, communicate desires, and initiate or negotiate sexual interactions [24], agency was predominantly attributed to men, whereas women were more often positioned in passive roles. This asymmetry contributed to sexual dissatisfaction, particularly when women perceived themselves as objects of desire rather than active partners. In some cases, sexual activity was employed strategically, as a means of resolving conflict, sustaining intimacy, or preventing extramarital involvement, thereby underscoring its broader relational functions. These findings suggest that the absence of reciprocal sexual agency may hinder couples’ ability to co-construct mutually satisfying sexual experiences, while simultaneously illustrating the pervasive influence of gendered expectations on intimate dynamics.

Another dimension of sexual agency identified in this study was controlling sex which encompassed sexual initiative, mutual attunement to sexual emotions, and active engagement within sexual relationships. Many women reported that sexual activity was expected to be initiated by their husbands, a dynamic that fostered feelings of objectification and, in some cases, sexual dissatisfaction. Consistent with this finding, prior research has demonstrated that women’s

experiences of self-objectification in sexual interactions are positively associated with body shame and negatively associated with overall life satisfaction [25].

Another finding suggest that, within the study population, prevailing social norms positioned virginity as both a gendered role and a social obligation for women. This emphasis on the preservation of virginity may underpin broader taboos surrounding sexual matters, contributing to restrictions on accessing sexual information and inhibiting the open expression of sexual needs. From a psychological perspective, such norms appear to shape the development of sexual agency and sexual schemas differently in men and women. Specifically, sexual agency was predominantly construed as a male attribute, while sexual submissiveness and passivity were ascribed to women. The tension arising from these gendered roles and expectations was closely associated with experiences of sexual dissatisfaction, highlighting the interplay between cultural prescriptions and individual psychological well-being.

Together, these findings demonstrate that sexual satisfaction in conservative societies such as Iran is constrained by entrenched taboos, restrictive sexual scripts, and unequal gender dynamics, and the internalization of these cultural scripts fosters shame and inhibits open communication even within marriage; thus, sexual satisfaction should be understood not simply as an individual psychological outcome, but as a socially mediated phenomenon shaped by norms, discourses, and power relations, underscoring the importance of addressing sociocultural factors in interventions aimed at enhancing sexual well-being.

Limitations

Again, see this section. Refer to the texts and compare two kinds of studies: qualitative and quantitative!

This study has several limitations that should be acknowledged. First, the findings may not be fully applicable to communities with different social and cultural contexts, and further research is needed to examine the transferability of results. The qualitative design also restricts

generalizability, underscoring the need for future studies to quantitatively evaluate the relationship between the identified psychosocial factors and newlyweds' sexual satisfaction before developing health promotion programs.

Second, the recruitment of participants mainly from premarital counseling and healthcare centers may have biased the sample toward health-conscious and educated couples, limiting the representation of those who did not attend such services. The relatively small sample size and reliance on self-reported data, particularly given the sensitivity of sexual topics, may also have introduced bias.

Finally, the dynamics of joint interviews posed challenges. The presence of a female interviewer may have influenced male participants to moderate their disclosures due to cultural taboos. Although individual interviews with men were offered by male interviewers when requested, some men may still have been cautious in expressing their experiences, potentially contributing to early data saturation among male participants. These cultural and contextual influences should be considered when interpreting the findings.

Conclusion

Please, write down how these findings apply to health care providers. It is important! In the abstract section, you do it a little!

This study showed that the three issues of abstract norms of sexual issues, sexual schemas, and sexual agency were among the psychosocial factors related to sexual satisfaction in newlyweds. These three elements are subjects that individual internalize in their social interaction from childhood to after marriage. Furthermore, these results showed that the social taboo of sexual issues impacts the couples' sexual attitudes and behaviors by banning the acquisition of sexual knowledge and skills and creating a feeling of the sinfulness of sexual issues.

Authors' contributions

Zahra Toghiyani, Ashraf Kazemi and Mahbobeh Taebi were involved in study conception and design. Ashraf Kazemi and Fatemeh Mohammadi involved in data collection supervising and responsible for coordinating the study. Zahra Toghiyani and Fatemeh Mohammadi involved in data collection. Ashraf Kazemi and Zahra Toghiyani involved in data analysis. Zahra Toghiyani, Ashraf Kazemi and Mahbobeh Taebi wrote the first draft of the manuscript. All authors read and approved the final version of the manuscript.

References

1. Ebrahimkhani S, Nouri R. Azizi M. The Impact of Sexual Esteem, Sexual Consciousness and Sexual Satisfaction on Marital Satisfaction. World Family Medicine. 2019; 17(2): 29-39. https://doi.org/10.5742/mewfm.2019.93614

2. Apostolou M, Christoforou C. Partner's traits which motivate people to stay in an intimate relationship: An explorative analysis. Pers Individ Dif. 2021; 183: 111155. https://doi.org/ 10.1016/j.paid.2021.111155

3. Khakkar M, Kazemi A. The relationship between depression and sexual satisfaction: An equation model analysis. J Educ Health Promot. 2023; 12(1): 419. https://doi.org/10.4103/jehp.jehp_1609_1622

4. Kilic D, Armstrong HL, Graham CA. The Role of Mutual Masturbation within Relationships: Associations with Sexual Satisfaction and Sexual Self-Esteem. Int J Sex Health. 2023; 35(4): 495-514. https://doi.org/10.1080/19317611.2023.2237950

5. Belu CF, O'Sullivan LF. Predictors of infidelity among couples. J Sex Med. 2024; 21(4): 270-2. https://doi.org/10.1093/jsxmed/qdad165

6. Daneshfar F, Keramat A. Sexual dysfunction and divorce in Iran: A systematic review. J Family Med Prim Care. 2023; 12(3): 430-9. https://doi.org/10.4103/jfmpc.jfmpc_991_22

7. Mundahl SJ. The Emotional Divorce: How Your Divorce Can be a Path to Self-discovery and a Happier Life: 2023.

8. Lavner JA, Weiss B, Miller JD, Karney BR. Personality change among newlyweds: Patterns, predictors, and associations with marital satisfaction over time. Dev Psychol. 2018; 54(6):1172-85. https://doi.org/10.1037/dev0000491

9. Bancroft JHJ. Human sexuality and its problems. Elsevier Health Sciences; 2008.

10. Rouhparvar Z, Javadnoori M, Shahali S. Parents' approaches to sexuality education of their adolescent boys: a qualitative study in Ahvaz, Iran. Reprod Health. 2022; 19(1): 69. https://doi.org/10.1186/s12978-022-01367-0.

11. Sheikhansari N, Abraham C, Denford S, Eftekhar M. A qualitative assessment of the sexual-health education, training and service needs of young adults in Tehran. BMC Public Health. 2021; 21(1):1386. https://doi.org/10.1186/s12889-021-11371

12. Golabi F, Bagher Alizadeh Aghdam M, Hosseinzadeh E. The power structure in the family and sexual satisfaction of women. Journal of Woman and Family Studies. 2023; 1(4), 20–44. https://doi.org/ 10.22051/jwfs.2023.41142.2887

13. Creswell JW, Creswell JD. Research Design: Qualitative, Quantitative, and Mixed Methods Approaches. 2018; Sage, Los Angeles.

14. Chua BS, Mutang JA, Tan CC, Jiang L Cloyd J, Tanucan M. Associations between intimacy in relationships and marital satisfaction across gender and in different durations of relationship. Cogent Psychology. 2025; 12(3): 123–37. https://doi.org/10.1017/jrr.2025.15

15. Lindgren BM, Lundman B, Graneheim UH. Qualitative content analysis, framing the analytical process of inductive content analysis to develop a sound study design. Quality & Quantity. 2*025;* 59(2): 123–45. https://doi.org/10.1007/s11135-025-02220-9

16. Graneheim UH, Lundman B. Qualitative content analysis in nursing research: concepts, procedures and measures to achieve trustworthiness. Nurse Educ Today. 2004; 24(2):105-12. https://doi.org/10.1016/j.nedt.2003.10.001

17. Adler RH. Trustworthiness in Qualitative Research. JHL. 2022; 38(4): 598-602. 10.1177/08903344221116620

18. Burr V. Social Constructionism (4th ed.). 2024; Routledge.

19. Wiederman MW. Sexual script theory: Past, present, and future. In J.DeLamater R. Plante F. Handbook of the sociology of sexualities Springer International 2025; 7–22.

20. Sidamo NB, Kerbo AA, Gidebo KD, Wado YD. Exploring Barriers to Accessing Adolescents Sexual and Reproductive Health Services in South Ethiopia Regional State: A Phenomenological Study Using Levesque's Framework. Adolescent Health, Medicine and Therapeutics. 2024; 15: 45-61. https://doi.org/10.2147/ahmt.S455517

21. Waling A, Farrugia A, Fraser S. Embarrassment, Shame, and Reassurance: Emotion and Young People's Access to Online Sexual Health Information. Sex Res Soc Policy. 2023; 20(1): 45-57. https://doi.org/10.1007/s13178-021-00668-6

22. Alam P, Lin L, Thakkar N, Thaker A, Marston C. Socio-sexual norms and young people's sexual health in urban Bangladesh, India, Nepal and Pakistan: A qualitative scoping review. PLOS Glob Public Health. 2024; 4(2): e0002179. https://doi.org/10.1371/journal.pgph.0002179

23. Astle S, Anders KM, McAllister P, Hanna-Walker V, Yelland E. The Conceptualization and Measurement of Sexual Self-Concept and Sexual Self-Schema: A Systematic Literature Review. J Sex Res. 2023: 1-12. https://doi.org/10.1080/00224499.2023.2244937

24. Vanwesenbeeck I, Cense M, van Reeuwijk M, Westeneng J. Understanding Sexual Agency. Implications for Sexual Health Programming. Sexes. 2021; 2(4): 378-96. https://doi.org/10.3390/sexes2040030

25. Pecini C, Di Bernardo GA, Crapolicchio E, Stathi S, Vezzali L, Andrighetto L. Stop 25 at me! associations between men's partner-objectification and women's self-objectification, body shame and life satisfaction in romantic relationships. Journal of Community & Applied Social Psychology. 2022; 32(6):1047-60. https://doi.org/10.1002/casp.2627

Table 1. Individual characteristics of the participants

Number	Age	Gender	Educational level	Duration of marriage (month)
1	25	Woman	Bachelor degree	36
2	33	Man	Master degree	60
3	28	Woman	Bachelor degree	7
4	25	Woman	Bachelor degree	9
5	21	Woman	Diploma	9
6	32	Man	Diploma	12
7	28	Man	Bachelor degree	5
8	19	Woman	Diploma	12
9	24	Woman	Bachelor degree	12
10	33	Woman	Bachelor degree	15
11	37	Woman	Bachelor degree	16
12	24	Woman	Bachelor degree	5
13	28	Woman	Master degree	17
14	33	Woman	Philosophy doctor	60
15	32	Woman	Bachelor degree	12
16	33	Man	Philosophy doctor	12
17	30	Woman	Bachelor degree	13
18	27	Man	Master degree	12
19	24	Woman	Master degree	48
20	32	Woman	Master degree	24
21	32	Man	Master degree	48
22	32	Woman	Master degree	36
23	34	Woman	Bachelor degree	48
24	37	Woman	Bachelor degree	12
25	44	Man	High school	15
26	34	Woman	Diploma	15
27	31	Man	Diploma	60
28	38	Man	Bachelor degree	48
29	31	Man	Bachelor degree	24
30	26	Man	Diploma	12
31	38	Man	Diploma	24

Table 2: Main and subcategories

Main Categories	Subcategories	Main codes
Social norms of sexual issues	Taboo of sexual self-disclosure	Embarrassment about obtaining sexual information
		Perceiving the sinfulness of sexual needs
		Embarrassment about sexual demands
	Social norms of meeting sexual needs	Sinfulness of sexual thoughts
		Sinfulness of masturbation
		Value of sexual restraint
	Obligation to preserve virginity in women	Stigma of breaking the hymen before marriage
		Requirement of confirming virginity in sexual relations after marriage
		Feeling of being sexually threatened by men
Sexual schemas	Sexual fantasies	Sexual diversification
		Sexual fantasizing
		Seeking affection versus pleasure
	Attitude towards sexual issues	Attitudes toward sex
		Sexual expectations
		Sexual Obedience
	Sexual qualification	Sexual attractiveness
		Skills of sexual behavior
		Playing sexual roles
Sexual agency	Sexual awareness and skills	Sexual awareness
		Skill of expressing sexual needs and demands
		Skill of satisfying sexual needs
	Sex as a means of control	Means of resolving marital conflicts
		Prevention of extramarital relations
		Maintaining marital intimacy
		Sexual authoritarianism: a means of confirming authority

	Controlling sex	Activism versus passivity
		Mutual understanding of sexual emotions
		Active interaction in a sexual relationship

COREQU cheklist

Click here to access/download
Supporting Information
COREQU.docx

Click here to access/download
Supporting Information
Ethic.pdf
