# Supplementary material for: A qualitative exploration of the psychosocial factors of sexual satisfaction in Iranian newlyweds
Source: PLoS One. 2026 Aug 13;21(8):e0356277. doi: 10.1371/journal.pone.0356277 (PMC13472429; doi:10.1371/journal.pone.0356277)
Supplement: Supplementary file 2 [file pone.0356277.s005.pdf]

Dear Editor and Reviewers

We sincerely thank the reviewers for the thoughtful, constructive, and valuable comments on our manuscript. We highly appreciate the reviewers' careful evaluation and insightful suggestions, which have helped us improve the quality of the manuscript. We have carefully addressed all comments and, to the best of our ability, incorporated the suggested revisions throughout the manuscript. We hope that the revisions made adequately respond to the reviewer's concerns and that the revised version will meet with the reviewer's approval. The manuscript has been revised according to the suggestions. The point-by-point response to the comments have been presented as follows:

Reviewers' comments:

Reviewer #1:

This study explores the psychosocial factors related to sexual satisfaction among Iranian newlyweds using a qualitative approach. The topic is important and understudied, particularly within the Iranian sociocultural context, and the manuscript presents rich qualitative data that provide valuable insight into the lived experiences of participants regarding sexuality, relationships, gender expectations, and social norms. The study also addresses a sensitive topic that is often difficult to investigate in more conservative cultural settings, which adds to its potential contribution to the literature.

However, the manuscript would benefit from substantial revision to improve conceptual clarity, logical organization, methodological transparency, and writing quality. In particular, the Introduction and Discussion sections would benefit from clearer structure and stronger integration with the broader literature, while the Methods section would benefit from clearer organization of procedures, participant structure, and analytic descriptions. Several thematic labels in the Results section could also be refined for greater conceptual precision and alignment with the qualitative data. Additionally, the manuscript requires considerable editing for grammar, formatting, and overall readability in order to align more closely with PLOS ONE reporting and formatting standards. Below, I provide detailed comments organized by manuscript section.

Abstract

Comment: The abstract repeatedly refers to "psychosocial factors," but the term is not clearly defined. Because "psychosocial factors" can encompass a wide range of constructs depending on the context, I recommend briefly clarifying the term at its first mention by providing a few examples (e.g., cultural norms, sexual communication, gender expectations, or interpersonal dynamics related to sexual issues). This would improve conceptual clarity for readers unfamiliar with the topic area.

Response: The comment has been done **[Abstract: Background; page 2]**

Comment The following sentence is difficult to follow due to awkward sentence structure: "This qualitative study was conducted with a qualitative content analysis approach on 31 heterosexual newlyweds that less than two years had passed since started having sex." I recommend revising this sentence for clarity and readability. For example, the authors could more clearly state that the study involved 31 heterosexual newlyweds who had been sexually active for less than two years.

Response: The sentence has been changed to: This qualitative study was conducted with a qualitative content analysis approach. The study involved 31 heterosexual newlyweds who had been sexually active for less than two years. **[Abstract: Method; page 2]**

Comment: In the Methods portion of the abstract, some methodological details may be overly technical for an abstract format. Specifically, the statement: "The criterion of data saturation was that no new inferential codes emerged after being repeated at least five times" introduces terminology ("inferential codes") that is not explained and may not be necessary at the abstract level. This detail may be better reserved for the full Methods section.

Response: The sentence "*The criterion of data saturation was that no new inferential codes emerged after being repeated at least five times*." has been omitted.

Comment Similarly, the sentence "The collected data were analyzed using Graneheim and Lundman method" may benefit from either a brief clarification of the analytic approach or simpler wording. If space is limited, the authors could instead state that the data were analyzed using qualitative content analysis.

Response: The comment has been done. **[Abstract, method, page 2]**

Comment In the Results section of the abstract, it would improve clarity to specify what the "three main categories" represent. For example, the authors could indicate that these were the three main psychosocial themes or categories identified through the qualitative analysis.

Response: The sentence change to: "*Social norms of sexual issues, sexual schemas, and sexual agency. were the three main psychosocial categories identified through the qualitative analysis*." **[Abstract, results, page 2]**

Comment The Conclusion section would benefit from both grammatical revision and a clearer synthesis of the study's findings and implications. The sentence beginning with "The results indicated that a more accurate understanding…" appears incomplete and difficult to follow. More importantly, the conclusion does not sufficiently summarize the key findings identified in the study, despite the manuscript having already outlined several important psychosocial themes (e.g., social norms, sexual schemas, and sexual agency). Rather than reiterating that "a more accurate understanding" is needed, the conclusion could more clearly state how the present findings contribute to that understanding and how these identified themes may inform culturally sensitive sexual education, counseling, or intervention programs for newlyweds in Iran.

Response: The conclusion section changes to **"**The findings provide insight into the key psychosocial dimensions underlying sexual satisfaction among newly married couples in Iran. These findings provide a culturally grounded understanding of how social norms, sexual schemas, and sexual agency shape sexual experiences and may inform the development of culturally sensitive sexual education, counseling, and intervention programs aimed at promoting sexual well-being and marital adjustment." **[Abstract, Cinclusion, page 2]**

Introduction

Comment: The Introduction would benefit from a clearer conceptual structure and more coherent progression of ideas. At present, several paragraphs transition abruptly between individual, relational, and societal consequences of sexual satisfaction/dissatisfaction without sufficient conceptual linkage, making the overall argument difficult to follow.

In the opening paragraph, the manuscript states that "sexual satisfaction is a significant component of sexual health," but does not define sexual satisfaction itself. I recommend briefly defining how sexual satisfaction is conceptualized within the literature before discussing its importance. This would provide readers with a clearer conceptual foundation for the study.

Response: The comment has been done. **[Introduction, the first Paragraph, Page 3]**

Comment: The statement "the lack of sexual satisfaction threatens society's health" appears overly broad and insufficiently supported. The manuscript transitions very quickly from individual marital outcomes to societal-level consequences without adequately explaining the

connection or providing supporting citations. I recommend either substantially revising this statement to make the argument more precise and evidence-based or narrowing the discussion to more directly established relational and psychological outcomes.

Response: The sentence" However, the lack of sexual satisfaction threatens society's health by generating a complex network of social, physical, and psychological complications. "has been omitted. **[Introduction, the first Paragraph, Page 3]**

Comment: The first two paragraphs of the Introduction would benefit from clearer differentiation between sexual satisfaction and marital satisfaction, as the manuscript appears to move between these constructs interchangeably at several points. Because these are related but distinct constructs, the relationships between them should be explained more explicitly and consistently throughout the Introduction.

The discussion of consequences also shifts abruptly across levels of analysis (e.g., individual psychological outcomes, marital outcomes, and broader social implications) without clear transitions. For example, the manuscript moves from depression and self-esteem, to "social difficulties," and then back to emotional divorce within the same section. Reorganizing these arguments and grouping consequences more coherently would substantially improve readability and conceptual clarity.

Response: The introduction reorganized according to the comment**. [Introduction, Page 3-4]**

Comment: The Introduction would benefit from a clearer concluding rationale explaining why studying sexual satisfaction is important, particularly in newlywed couples and within the Iranian sociocultural context. Currently, the early paragraphs discuss various consequences but do not clearly synthesize why this issue warrants the present investigation.

The paragraph beginning "The adverse and significant effects of sexual dissatisfaction…" relies heavily on a single cited study regarding early marriage and marital satisfaction. I recommend incorporating a broader and more diverse body of literature, including studies from multiple cultural and national contexts, to better establish the importance of sexual satisfaction during the early years of marriage. Additionally, the direction and nature of these associations should be described more explicitly (e.g., whether greater sexual satisfaction is associated with higher marital satisfaction, relationship stability, or quality of life).

Response: The sentences "Decreased marital satisfaction can increase the probability of depression [3], and decreased self-esteem [4], and sexual dysfunction" have been omitted.

Comment: The paragraph discussing “the cycle of sexual response” and environmental factors appears conceptually disconnected from the surrounding discussion. The transition from physiological sexual response, to sexual pleasure, to environmental influences and biopsychosocial framing is difficult to follow. I recommend either substantially revising this paragraph for clarity or integrating the biopsychosocial framework more naturally into the broader literature review.

Response: The paragraph “Although the cycle of sexual response, followed by sexual pleasure, goes through a physiological …… information” has been deleted.

Comment: More generally, the Introduction could be strengthened by improving the transition into the Iranian sociocultural context. Rather than introducing the Iranian context somewhat abruptly, the authors may consider first summarizing broader international evidence regarding sexual satisfaction and marital outcomes, and then explaining why the Iranian context may differ due to distinct sociocultural norms surrounding sexuality, gender, and marriage.

The discussion of Iranian sociocultural factors would benefit from greater specificity and clearer explanation. For example, the manuscript states that “sociocultural constraints and inadequate knowledge are key challenges,” but does not sufficiently explain what these constraints are or how they influence sexual satisfaction and communication among newlyweds.

Response: The comment has been done. **[Introduction, the first Paragraph, Page 4]**

Comment: Several sentences in the Introduction are conceptually unclear and would benefit from revision for clarity and precision. For example, the phrase “the development of sexual concepts as a cognitive generation about sexual aspect” is difficult to interpret. Similarly, statements regarding women’s and men’s attitudes being formed in a “sociocultural context, which has a dissimilar view on sexual issues” are vague and should more explicitly describe the relevant cultural differences or expectations being referenced.

Response: Due to the opinion of the respected referee and one of the referees regarding the length of the introductory sentence " Compared to modern countries, in traditional societies such as Iran, the development of ….. dissimilar view on sexual issues. " has been deleted.

[Introduction, the first Paragraph, Page 3]

Comment: The rationale for conducting the study in Iran is currently spread across multiple paragraphs with overlapping ideas, resulting in some redundancy and reduced clarity.

Consolidating and reorganizing these sections would strengthen the overall narrative and improve the logical flow of the Introduction.

Response: The comment has been done. **[Introduction, the first Paragraph, Page 3-4]**

Comment: The justification for the qualitative design and the focus on newlyweds could also be integrated more cohesively. For example, the authors could first explain why the early years of marriage represent a particularly formative phase for sexual and relational development, then argue that the limited existing evidence and sociocultural specificity of the topic make a qualitative approach especially appropriate for capturing lived experiences and identifying emergent psychosocial themes.

The final research question is generally appropriate and relevant to the aims of the study. However, the preceding rationale could more clearly build toward this question through a more logically organized review of the literature and clearer articulation of the study gap.

Response: The comment has been done **[Introduction, the first Paragraph, Page 4]**

Methods

Comment: The "Researcher Characteristics and Reflexivity" section appears closer to a positionality or reflexivity statement than a traditional methodological subsection. While reflexivity is important in qualitative research, the authors should ensure that this section aligns with the journal's reporting standards and qualitative reporting guidelines.

Response: The subsection "*Researcher Characteristics and Reflexivity*" has been omitted and the comment has been done**. [Page 5-7, Method**]. Also, some unnecessary reports have been omitted.

Comment: The participant description would benefit from clearer demographic reporting. Although participant-level information is later presented in Table 1, the main text should also summarize key demographic characteristics such as age ranges or mean ages, educational background, and other relevant sample characteristics. In addition, the manuscript does not clarify whether participants were ethnically homogeneous or whether there was diversity in ethnicity or cultural background, which may be relevant to interpretation of the findings.

Response: The comments have been done. [**Page 5; method/Participants subsection]**

Comment: The inclusion and exclusion criteria require clarification. Specifically, the sentence regarding pregnancy and infertility is difficult to follow, and it is unclear whether pregnancy and infertility were treated as exclusion criteria because of their potential influence on sexual satisfaction. This should be stated more explicitly.

Response: The comments have been done. [**Page 5; method/Participants subsection]**

Comment: The description of data saturation remains somewhat unclear. The statement that "no new inferential codes emerged after being repeated at least five times" would benefit from additional clarification or simpler wording, as the concept of "inferential codes" is not sufficiently explained for readers unfamiliar with the analytic framework.

Response: The sentence **"**Data collection continued until no new data emerged.**"** has been replaced.

Comment: The manuscript states that purposive sampling aimed to maximize diversity in age, education, and duration of marriage/sexual activity, and these characteristics are shown in Table 1. However, the text does not explicitly direct readers to the table or summarize these characteristics in the narrative. Including brief summary statistics and clearer reference to the table would improve readability and transparency.

Response: The comment has been done. **[Method/ page 5]**

Comment: The overall structure and chronology of the "Procedure and Data Collection" section would benefit from clearer organization. Although the section contains substantial methodological detail, the sequence of information is somewhat difficult to follow, particularly regarding participant recruitment, interview procedures, and the progression from preliminary interviews to the main study interviews.

Response: Some unnecessary reports have been omitted.

Comment: It is currently unclear how participants were assigned to or selected for individual versus joint interviews. The manuscript would benefit from explicitly describing the rationale and procedure for determining whether participants completed individual interviews, couple interviews, or both.

Response: The sentence "In total, 21 individual interviews and 5 couple interviews were conducted during the data collection process." has been mentioned. **[Method/ Procedure and Data collection; Page 6]**

Comment: Relatedly, the participant structure of the study is somewhat ambiguous. Initially, the manuscript gives the impression that the study focused on newlywed couples as dyads. However, the Methods later suggest that some participants were interviewed individually without their spouses participating, whereas others participated in couple interviews. This distinction should

be clarified more explicitly so that readers can better understand the sample composition and analytic structure.

Response: Because the research population consisted of young couples and sampling was done among them, the phrase " newlywed couples " was used. However, for greater clarity, the sentence "In total, 21 individual interviews and 5 couple interviews were conducted during the data collection process" was added. **[Method/ Procedure and Data collection; Page 6]**

Comment: The section describing the development of interview questions appears to indicate that preliminary interviews were conducted to refine the interview guide. This process is appropriate, but the procedural description could be streamlined and clarified. For example, the authors could more directly explain that pilot or preliminary interviews were used to develop and refine the guiding questions for the main study.

Response: The comment has been done and some unnecessary reports have been omitted**.**
[Method, page 6]

Comment: The content of the interview procedures is generally thorough and appropriate, including the use of guiding questions, probing questions, confidentiality procedures, and interviewer considerations. However, the presentation would benefit from improved organization. For example, information about interview duration, interview settings, data collection dates, confidentiality procedures, and interviewer characteristics could be grouped more systematically to improve readability and methodological transparency.

Response: According to the comment and the comment of another reviewer, the method section has been reorganized and some unnecessary reports have been omitted.

Comment: The Data Analysis section is generally well developed and clearly described. The stepwise explanation of coding, category development, and thematic interpretation is a strength of the manuscript. However, the manuscript does not specify whether any qualitative analysis software or coding platforms were used during data management or analysis (e.g., NVivo, MAXQDA, Atlas.ti, or manual coding only). If software was used, this should be stated explicitly. If coding was conducted entirely manually, the authors may also wish to clarify this.

Response: At the first line of the data analysis subsection this point (manual analysis) was mentioned. **[Method, page 7]**

Comment: The “Trustworthiness” section contains relevant and important information regarding credibility, dependability, transferability, and confirmability. However, the authors may consider

integrating some of this discussion more directly into the broader methodological or data analysis sections rather than presenting it as a fully separate subsection, depending on journal style and qualitative reporting conventions.

Response: We thank the reviewer for this valuable comment. As the Methods section is already relatively extensive, we would respectfully suggest, if deemed appropriate by the reviewer, presenting the Trustworthiness criteria as a separate subsection with its own heading. We believe this format would improve the overall clarity and readability of the manuscript.

Results

Comment: At the beginning of the Results section, the manuscript reports the sample sizes and mean ages of participants. However, it would improve reporting transparency to also include measures of variability such as standard deviations and/or age ranges in either the text or Table 1.

Response: The comment has been done. **[Results; page 8]**

Comment: Overall, the Results section contains rich and meaningful qualitative data, and the participant quotations provide valuable insight into the lived experiences of Iranian newlyweds. The thematic findings are generally interesting and potentially important. However, some of the category and subcategory labels could be refined to improve conceptual precision and alignment with the supporting examples. For example, the subcategory “The taboo of sexual self-disclosure before marriage” may not fully capture the content of the participant quotations, which largely focus on the inability or social prohibition against discussing sexual topics within families and social contexts. The authors may wish to consider alternative wording emphasizing the taboo surrounding sexual discussion or communication before marriage.

Response: At the end of the narration, the phrase that we had omitted before was added. **[Results; Page 8, the last line]**

Comment: Similarly, the subcategory “Social norms of meeting sexual needs before marriage” could potentially be revised for conceptual clarity. Based on the examples provided, the emphasis appears to be less on meeting sexual needs and more on the suppression, restraint, or moral regulation of sexual thoughts and desires prior to marriage. Revising the wording to better reflect this underlying theme may improve interpretability.

Response: Social norms of meeting sexual needs before marriage has been change to “Social norms of restraint sexual desires” **[Results, page 8]**

Comment: The broader category "Sexual schemas" is conceptually interesting and appears relevant to the findings. However, some of the participant examples also involve the influence of pornography, media representations, and imagined expectations regarding sex prior to actual sexual experience. The authors may wish to consider whether these elements could be more explicitly reflected in the wording or interpretation of this category.

Response: Thank you for this insightful comment. As this study employed a concept analysis approach, our primary focus during data analysis was on describing and interpreting participants' experiences related to the concept under investigation rather than examining the sources of those expectations in depth.

Comment: Within the subsection "Attitude toward sexual issues," some overlap appears to exist between the examples used here and the earlier discussion of sexual taboos and restrictions on sexual communication before marriage. The distinction between these categories could therefore be clarified further to strengthen conceptual separation between themes.

Response: The sentence "Attitudes towards sex' was concluded from narratives that reflected individuals' fell about sexual issues" has been changed to "Attitudes towards sex' was concluded from narratives that reflected individuals' approach about sexual issues". Also the sentence "These main cods were extracted from participants' statements and indicated the taboo of social issues in society that caused the participants to consider sexual issues before marriage as immoral and feel embarrassed to express their need for sexual information. " has been changed to "These main cods were extracted from participants' statements and indicated the taboo of social issues in society that caused the participants to feel embarrassed to express their need for sexual information.". **[Results, page 11]**

Comment: The term "Sexual obedience" may benefit from reconsideration, as it may not fully capture the relational dynamics reflected in the participant narratives. The examples appear to relate more closely to themes such as sexual obligation, marital duty, or perceived responsibility to satisfy a spouse sexually. The authors may wish to consider alternative terminology that more accurately reflects these experiences.

Response: the phrase "Sexual obedience" has been changed to "Sexual obligation" **[Results, page 11]**

Comment: Within the category "Sexual agency," there appears to be some conceptual overlap between "Sexual awareness and skills" and earlier themes such as "skills of sexual behavior"

under “Sexual qualification.” The distinctions between these categories could be clarified further to improve thematic coherence.

Response: Skills of sexual behavior change to Interactive sexual behavior and Sexual awareness and skills has been changed to Interactive sexual behavior and sexual awareness. **[Results, page 12]**

Comment: The subcategory “Sex as a means of control” may not fully align with all of the included examples and codes. Some examples appear to relate more to conflict resolution, maintenance of intimacy, relational negotiation, or prevention of relational insecurity rather than “control” per se. The authors may wish to reconsider the wording of this category to better capture the broader relational functions reflected in the data.

Response: The phrase “Sex as a means of control” has been changed to “Sex as a means of conflict resolution”. **[Results, page 13]**

Similarly, the subcategory “Controlling sex” may not fully reflect the participant quotations or thematic content. The included themes (e.g., activity versus passivity, mutual understanding, active participation) appear more related to relational or interpersonal dynamics within sexual interactions. Revising this label may improve conceptual clarity and alignment with the data.

Discussion

Comment: The Discussion section would benefit from beginning with a clearer reminder of the study aims and a concise summary of the principal findings before moving into interpretation. This would help orient readers and provide a stronger conceptual framework for the subsequent discussion.

Response: The comment has been done. **[Discussion, page 14]**

Comment: Early in the Discussion, the manuscript refers to “dominant cultural narratives, religious traditions, and gendered expectations” shaping sexual experiences in Iran. While this interpretation is reasonable, the discussion would benefit from briefly elaborating on what these narratives and expectations entail in the Iranian sociocultural context, particularly for international readers who may be less familiar with these dynamics.

Response: The comment has been done. **[Discussion, page 14]**

Comment: The discussion of social taboos surrounding sexuality is generally strong and well connected to the qualitative findings. However, when comparing the present findings to previous studies, the manuscript could more thoroughly contextualize the cited literature. For example,

when stating that “other studies reported that in some societies, the taboo of sexual issues has limited the acquisition of information from reliable sources,” the authors could elaborate on where these studies were conducted, how those societies compare culturally to Iran, and what specific sociocultural or religious factors may contribute to these similarities or differences.

Response: The comment has been done. **[Discussion, page 14, 15]**

Comment: More broadly, the Discussion section would be strengthened by placing the findings more consistently within the wider international literature. While the manuscript provides interpretation of the themes, there is comparatively less discussion of how these findings align with, extend, or differ from studies conducted in other cultural settings, including both culturally similar and culturally distinct contexts. The sections discussing sexual schemas, sexual qualification, and sexual agency summarize the study findings effectively, but would benefit from deeper engagement with prior literature. For example, the authors could discuss whether similar patterns regarding gender roles, expectations, media influences, or relational dynamics have been observed in other conservative, collectivist, or more individualistic cultural contexts.

Response: The comment has been done. **[Discussion, page 15]**

Comment: The paragraph revisiting virginity and its influence on sexual agency and sexual schemas raises important points, particularly regarding how cultural expectations surrounding virginity shape gendered sexual development. However, the organization of this portion of the Discussion could be improved so that the conceptual connections between virginity norms, sexual schemas, and sexual agency are more explicitly linked and integrated with the preceding discussion.

Response: The comment has been done. **[Discussion, page 16]**

Comment: The manuscript would benefit from a more explicit discussion of the practical implications of the findings. Although the Abstract briefly mentions implications for premarital counseling and educational programs, these applications are not sufficiently developed within the main Discussion section. The authors could elaborate on how the identified psychosocial themes may inform culturally sensitive sexual education, premarital counseling, communication training, or couple-based interventions for newlyweds in Iran.

In addition to practical implications, the Discussion could more explicitly address directions for future research. For example, the findings could inform future quantitative studies examining the

prevalence, structure, or associations of the identified psychosocial themes in larger and more diverse samples.

Response: The comment has been done. **[Discussion, page 17]**

Comment: The Conclusion section would benefit from clearer alignment with the rest of the manuscript. For example, the manuscript refers to “three issues” in the Conclusion, whereas earlier sections consistently describe these as “three categories” or themes. Maintaining consistent terminology throughout would improve clarity.

Response: The comment has been done. **[Discussion, page 19]**

Comment: The Conclusion could also more effectively synthesize the aims, findings, and implications of the study. Rather than primarily restating the existence of social taboos and related psychosocial factors, the conclusion could briefly reiterate the study purpose, summarize the key identified themes, and emphasize how these findings contribute to understanding sexual satisfaction among Iranian newlyweds and may support future research, counseling approaches, and educational initiatives.

Response: The comment has been done. **[Discussion, page 19]**

Comment: More generally, while the Discussion provides substantial interpretation of the findings, it would benefit from greater emphasis on how the study contributes constructively to addressing the identified issues. In its current form, some sections primarily restate the presence of sociocultural barriers without sufficiently discussing how the findings may help guide future interventions, support systems, educational programs, or broader conversations surrounding sexual health and communication in culturally sensitive contexts.

Response: We sincerely thank the reviewer for this insightful and constructive comment. In revising the Discussion section, we carefully considered and incorporated the reviewer’s suggestions, with particular attention to strengthening the emphasis on the constructive implications of the findings. Efforts were made to move beyond merely describing sociocultural barriers and to more explicitly highlight how the results can inform future interventions, educational programs, support systems, and culturally sensitive approaches to sexual health and communication.

Manuscript Presentation and Writing Quality

Comment: The manuscript would benefit from careful revision for grammar, writing clarity, and overall language consistency. Throughout the paper, there are numerous sentences with awkward phrasing, unclear wording, grammatical inaccuracies, inconsistent terminology, and occasional missing punctuation marks. While the core findings of the study are valuable, substantial language editing would improve readability and clarity.

Response; The manuscript has been edited by an English editor.

Comment: The manuscript would also benefit from more careful attention to formatting and journal style requirements. I recommend that the authors carefully review the PLOS ONE author guidelines to ensure that the manuscript conforms to the journal's formatting standards.

Several formatting inconsistencies were noted throughout the manuscript, including inconsistent line spacing, paragraph spacing, reference formatting, numbering, and spacing conventions. Greater attention to stylistic consistency would improve the professionalism and readability of the submission.

The manuscript also contains highlighted text in multiple sections. It is unclear whether these highlights are intended for editorial purposes or were unintentionally retained in the submitted version. The authors should ensure that all editorial markings or tracked formatting elements are removed prior to resubmission.

Given the frequency of grammatical and stylistic issues throughout the manuscript, the authors may wish to consider professional English-language editing or manuscript editing services prior to resubmit

Response; The manuscript has been revised according to the journal's formatting standards.

R3

Comment

In introduction section, you should discuss the specific perspective on these issues. For example, quality of marital life, emotional divorce, or a consultation program for ne
wlyweds. Social and cultural factors would affect sexual behaviors. All of the epidemiologic stud ies showed and stressed the social factors on health behaviors. These are
clear in past studies and the majority of epidemiologic studies. I suggest " consultation program ming" before and after marriage, you choose. It would be an important perspective and applicabl e for healthcare providers.

Response: We thank the reviewer for this valuable comment and agree that a substantial body of literature has already documented various determinants of sexual satisfaction. The challenges and gaps that exist for the necessity of conducting research for effective planning were explained. **[Page 4, Paragraph 3]**

Comment: Accordingly, examining how socially and culturally embedded beliefs are internalized and influence sexual satisfaction remains highly relevant. Furthermore, a notable strength of the present study is its couple-centered perspective. By including both husbands and wives, the study was able to capture relational and interpersonal dimensions of sexual satisfaction and identify psychosocial factors that emerge through the interaction of partners, rather than solely through individual experiences. We have clarified these points in the revised manuscript.

Response: The comment has been done **[page 3, the last paragraph]**

Comment: Really? Are you sure it can be conducted as an empirical study on these factors? it is not suitable for your study, and here. (Despite the importance of this issue, there remains a scarcity of empirical research exploring the intersection of psychosocial factors and sexual satisfaction within Iranian society.)

Response: the sentence Despite the importance of this issue, there remains a scarcity of empirical research exploring the intersection of psychosocial factors and sexual satisfaction within Iranian society.) has been omitted.

Comment: your study is qualitative, so you can't judge the "effect" of those factors on satisfaction.

Response: The study question has been corrected**. [Page 4 the last paragraph]**

Comment: Here, draw the characteristics of participants (sex, age, etc.) in a table.) (Sampling)

Response: The comment has been done **[Method: Sampling, Page 5; paragraph 3]**

Comment: This section (Procedure and Data collection) would be summarized. You should omit the repeated sentences, and you should stress the important points that are necessary for this section.

Response: The Procedure and Data collection subsection has been revised and reorganized.

Comment: In this section, you should, based on Grranheim & Lundman's method, present the steps of analysis. Also, you would be designing the table with quotations, codes, and categories. In other words, readers could understand how you do the steps of analysis.) (Data analysis)

Response: The comment has been done **[Method/ Data analysis /Page 7]**

Comment: Set it in the text, in the participant section. (results: presented in Table 1)

Response: The comment has been done.

Comment: This section should be based on the " problem statement".Because of my opinion in that section, I suggest regulating these findings. Your discussion is based on evidence that was known. So, I stress my idea in the first section that I mentioned above. (Discussion)

Response: In response to this and other reviewers' comments, the Introduction was substantially reorganized and the Discussion was strengthened to better contextualize the findings, clarify the study's contribution, and highlight their implications for future research and practice.

[Discussion, Page 17]

Comment: Please, write down how these findings apply to health care providers. It is important! In the abstract section, you do it a little!

Response: In the discussion section and conclusion the practical implications has been mentioned. **[Discussion, Page 17]**

Finally, we would like to express our sincere gratitude to the reviewers for their thoughtful and constructive comments. Their valuable insights have greatly enriched the manuscript and have helped us enhance its scientific quality and presentation. We deeply appreciate the time, effort, and expertise invested in reviewing our work.
