# Supplementary material for: A qualitative exploration of the psychosocial factors of sexual satisfaction in Iranian newlyweds
Source: PLoS One. 2026 Aug 13;21(8):e0356277. doi: 10.1371/journal.pone.0356277 (PMC13472429; doi:10.1371/journal.pone.0356277)
Supplement: Supplementary file 3 [file pone.0356277.s006.pdf]

Dear Editor and Reviewers

We are thankful for the comprehensive review and insightful suggestions for the manuscript entitled "A qualitative exploration of the psychosocial factors of sexual satisfaction in Iranian newlyweds". The manuscript has been revised according to the suggestions. The point-by-point response to the comments have been presented as follows:

Journal Requirements:

If the reviewer comments include a recommendation to cite specific previously published works, please review and evaluate these publications to determine whether they are relevant and should be cited. There is no requirement to cite these works unless the editor has indicated otherwise.

Please review your reference list to ensure that it is complete and correct. If you have cited papers that have been retracted, please include the rationale for doing so in the manuscript text, or remove these references and replace them with relevant current references. Any changes to the reference list should be mentioned in the rebuttal letter that accompanies your revised manuscript. If you need to cite a retracted article, indicate the article's retracted status in the References list and also include a citation and full reference for the retraction notice.

Response: We clarify that the reference list has not been modified. In addition, we carefully re-checked all references to verify their retraction status. We confirm that none of the cited references has been retracted.

Comments to the Author

Reviewer #3: Minor/moderate points:
- The manuscript would benefit from further language editing and proofreading (e.g. in the abstract: "Psychosocial factors related to sexual issues […] related to sexual issues", "This qualitative study was conducted with a qualitative content analysis approach. […] The data were analyzed using qualitative content analysis."; "interviews conducted either individually or in dyadic settings. conducted either individually or in dyadic settings"; results, "cods", "One of the man participants", "One of the woman participants")

Response: Thank you for your valuable comment. We carefully proofread the entire manuscript and revised the language to improve clarity, readability, and grammatical accuracy. Specifically, redundant wording and repeated phrases were removed, typographical errors (e.g., cods → codes) were corrected, and grammatically incorrect expressions. We also revised the abstract and other relevant sections to eliminate repetitive expressions. **[Abstract, page 1; Results: page 9-11]**

- Abstract, “Consolidated Criteria for Reporting Qualitative Research guidelines”: Please provide the mentioned guidelines (e.g. in a table) in the main text or supplement.

Response: We have revised the Methods section to explicitly state that the study was reported in accordance with the Consolidated Criteria for Reporting Qualitative Research (COREQ) guidelines. In addition, we have included the completed COREQ checklist as a supplementary file, indicating where each reporting item is addressed in the revised manuscript. **[Sup 1]**

- Abstract, “The data were collected and analyzed through individual and joint semi-structured interviews.”, and paragraph headed “Participants” in the “Methods and Materials” section: As already mentioned in the previous reviews, the cited sentence is not entirely clear, as it could mean that all participants went through both individual and joint interviews. Instead, it should be clarified that part of the sample consisted of individuals interviewed without their partner, and also couples interviewed jointly, while also mentioning the exact numbers for both in the abstract. In the “Participants” paragraph, it should also be clarified that some were interviewed individually and others in a joint interview (while also mentioning the exact numbers of participants for both) – this information is provided later (at the end of the subsection “Procedure and Data collection”), but should be clear earlier.

Response: We agree that the original wording could have led to the misunderstanding that all participants underwent both individual and joint interviews. To eliminate this ambiguity, we have revised the Abstract and the “Participants” subsection of the Methods to clearly specify the interview format and the exact numbers. Specifically, we clarified that a total of 36 semi-structured interviews were conducted, comprising 31 individual interviews (19 women and 12 men) and 5 dyadic interviews involving 5 couples (10 participants). We also clarified that the dyadic interviews were conducted with couples whose partners had previously participated in individual interviews. These revisions improve the clarity and transparency of the study design. **[Abstract: page 2; Method: page 7, paragraph 3]**

- In the first sentences of the introduction, the focus is set to married couples and marital satisfaction only. However, the research questions and findings may equally apply to couples in a committed romantic relationship without being married. Hence, I would either explain the direct focus on married couples only, or start off a little broader considering couples in committed romantic relationships in general.

Response: We appreciate the reviewer's suggestion. The focus on married couples was intentional because the study was conducted in a sociocultural context where intimate and sexual relationships are socially, legally, and religiously recognized only within marriage. Therefore, broadening the scope to include unmarried couples would not be consistent with the cultural and ethical context in which the study was designed and approved. To clarify this rationale, we have revised the Introduction by explicitly explaining that the importance of investigating sexual satisfaction among married couples should be understood within such sociocultural contexts. **[Page 3, First paragraph]**

- In the introduction and in the discussion, biological variables are mentioned without further

explanation or definition (“sexual satisfaction is a multidimensional construct that extends beyond biological functioning”, “sexual experiences were not merely individual or biological but socially constructed”, “Sexual education programs in conservative contexts such as Iran should go beyond biological information”). Please clarify what kind of biological factors are meant here, by shortly defining and possibly also explaining how they are relevant.

Response: Accordingly, we have revised the relevant statements in the Introduction and Discussion by briefly defining biological factors as aspects of sexual functioning, including sexual desire, arousal, physiological sexual responses, and, where relevant, reproductive anatomy and physiology. **[Page 3, paragraph 4]**

- Abstract, Methods: It is stated that "sampling was implemented using a purposeful method and continued until data saturation." However, there are no details on how saturation was assessed or at which point it was reached. Please provide a short description of the criterion used to determine saturation (e.g. no new codes emerging across successive interviews), and an indication of approximately when saturation was reached relative to the total sample. The paper also notes that "early data saturation among male participants" may have occurred due to cultural constraints on male disclosure — but if saturation was reached early for men, this raises a serious question about whether men's experiences are adequately represented in the findings. A sample of 12 men is already skewed relative to 19 women; if saturation was reached early due to underrepresentation of male perspectives rather than genuine thematic exhaustion, the findings may systematically underrepresent men's experiences, which should be discussed.

Response: Methods section to provide additional details regarding both purposive sampling and data saturation. In the Participants subsection, we clarified how purposive sampling was implemented to recruit participants with diverse characteristics and experiences relevant to the study aim. In the Procedure and Data Collection subsection, we explained that data collection and analysis were conducted concurrently and that sampling continued until successive interviews yielded no new codes or concepts, indicating that data saturation had been achieved. Also, we agree that the original wording could have implied that early data saturation among male participants reflected comprehensive thematic coverage. To avoid this interpretation, we have revised the Limitations section to clarify that the apparent early saturation may have resulted from restricted disclosure due to sociocultural factors rather than complete exploration of men's experiences. We also explicitly acknowledge that this may have led to the underrepresentation of men's perspectives in the findings and that this limitation should be considered when interpreting the results. [Page 5: last line & Page 6 2 first lins]

- Methods, Procedure and Data collection, “In order to prevent the bias of the spouse’s presence in couple interviews, an attempt was made to deepen the interviews with questions such as “What do you think of your spouse’s statements?””: The sentence does not quite make sense conceptually. Asking “What do you think of your spouse’s statements?” does not really prevent bias from the spouse’s presence. Instead, it draws attention to the spouse’s statements and may even increase social desirability or conformity pressure. Please clarify if this is a phrasing error in describing the procedure or whether the interviews were actually conducted this way. If the latter, potential implications for results from the interviews should be discussed critically.

Response: This was a phrasing error in the description of the procedure rather than in the conduct of the interviews. Accordingly, we have revised the sentence to read: "Could you elaborate on what your spouse has just said?" to accurately reflect the follow-up question used during the dyadic interviews. **[Page 7, Paragraph 2]**

- Results, “Of the 34 eligible individuals invited for the study, 31 individuals […]”: Please clarify what happened to the remaining 3 participants.

Response: We have added this information to the Participants section and clarified that the remaining three eligible individuals declined to participate. **[page 8, result, paragraph 4]**

- Several category labels are questionable from my point of view. “Social norms of sexual issues” could rather be “Social norms surrounding premarital sexuality”, “Premarital sexual norms”, or “Social norms regulating premarital sexual behavior”. Instead of “Sexual qualification”, “Sexual self-presentation”, “Perceived sexual competence”, or “Sexual attractiveness and competence” may be more suitable. Instead of “Sexual agency”, please consider “Sexual agency and control” or “Sexual power and control”. Please consider changing the labels throughout the manuscript.

Response: We sincerely thank the reviewer for this thoughtful and constructive suggestion. The category of “Social norms of sexual issues” has been changed to “Premarital sexual norms”. Sexual qualification has been changed to “Perceived sexual competence”, Sexual agency” has been changed to “Sexual agency and control” **[results section and table 3 and any related sentences in discussion]**

-Also, it appears the subcategory labels in the subsection “sexual agency” do not match (e.g. “sexual knowledge and skills” versus “sexual awareness and skill”).

Response: We agree that consistency between category and subcategory labels is important. In our analysis, however, sexual knowledge and skills was conceptualized as a fundamental component of sexual agency rather than as an independent construct. Sexual agency reflects an individual's capacity to make informed decisions, communicate needs and preferences, and act intentionally in relation to sexuality. Such agency is inherently supported by adequate sexual knowledge and the skills required to understand, evaluate, and navigate sexual situations. Therefore, we believe that the subcategory is conceptually aligned with the overarching category, as it represents one of the essential psychological foundations through which sexual agency is developed and expressed. Nevertheless, we have carefully reviewed the wording throughout the manuscript to ensure greater consistency and clarity between the category and its subcategories.

- In some Discussion sections I would consider adding relevant citations to better connect this study’s findings with earlier research, for example in the paragraph beginning with “These psychological factors”.

Response: Our intention in this paragraph and others were to interpret the psychosocial factors identified in the present study rather than to make a general statement about psychosocial factors

reported in the broader literature. To avoid this potential ambiguity, we have revised the wording to explicitly refer to the psychosocial factors identified in our study. **[page 15: paragraph 3]**

- Discussion, “Consistent with social constructionist perspectives, sexual experiences were not merely individual or biological but socially constructed through dominant cultural narratives, religious traditions, and gendered expectations [22].”: Here it is unclear whether the finding (“sexual experiences were not merely individual or biological but socially constructed”) refers to this study or the citation (Burr, 2024) – please clarify; if the former please elaborate on what kind of individual, biological, and social factors are referred to here.

Response: We agree that the original wording could be interpreted as referring either to the findings of the present study or to the cited literature. To improve clarity, we revised this section to explicitly distinguish between our interpretation of the findings and the existing theoretical perspective. Specifically, we introduced the social constructionist perspective as an established theoretical framework and clarified that the statement regarding the social construction of sexual experiences reflects the existing literature (Ref. 22), which provides a conceptual basis for interpreting our findings. This revision also improves the logical flow between the discussion of previous evidence (Ref. 21) and the theoretical explanation offered by social constructionism (Ref. 22) **[page 15: last paragraph, page 16: first paragraph]**

- The conclusion is still very brief without adding any integrative insights. Please enrich the conclusion by shortly stating what the three categories collectively reveal about sexual satisfaction as a socially mediated phenomenon in the Iranian context, potentially also stressing the single most important implication(s) for policy or practice.

Response: According to valuable suggestion, we have revised the conclusion to provide a more integrative interpretation of the three interrelated categories, emphasizing that sexual satisfaction among Iranian newlyweds is a socially mediated phenomenon shaped by the interplay of premarital sexual norms, sexual schemas, and sexual agency. We have also strengthened the practical implications by highlighting the importance of culturally sensitive sexual education, premarital counseling, and couple-based interventions that address restrictive social norms, promote healthy sexual schemas, and strengthen sexual agency. **[Page 20, Conclusion section]**

Reviewer #4: General comment

I would like to thank the authors for their careful and thorough revision. The manuscript has improved substantially since the previous version. The Introduction is more coherent, the methodological reporting has been strengthened, the Discussion has been expanded, and the practical implications are now better articulated. Overall, I believe the manuscript now makes a valuable contribution to the literature on sexual satisfaction in the Iranian sociocultural context. I have only a few minor comments that I believe would further strengthen the manuscript.

Comment 1: The three overarching categories ("Social norms of sexual issues", "Sexual schemas", and "Sexual agency") provide a clear and useful framework for organizing the findings. However, it remains unclear whether these categories were developed entirely inductively from the data or whether they were informed by existing theoretical concepts from

the literature. Given that notions such as sexual agency and sexual schemas are already well established in sexuality research, I encourage the authors to clarify their analytical approach and explain more explicitly how these categories emerged during the analytic process.

Response: We sincerely thank the reviewer for this insightful comment. As described in the revised Methods section, our analysis followed an inductive qualitative approach in which meaning units, codes, subcategories, secondary categories, and overarching categories were developed iteratively from the participants' narratives. Although concepts such as sexual schemas and sexual agency are well established in the literature, they were not used as predefined analytical categories. Rather, after the categories had emerged from the data, relevant theoretical concepts were consulted, where appropriate, to support their conceptual interpretation and naming. We have revised the Methods section to make this analytical process more explicit. **[Page 7, paragraph 5]**

Comment 2: One aspect that could further strengthen the manuscript would be to make the relationships between the three identified categories more explicit. Throughout the Discussion, the findings suggest that social norms shape sexual schemas, which in turn influence sexual agency and, ultimately, sexual satisfaction. Rather than presenting these categories primarily as parallel themes, the authors may wish to discuss their dynamic interrelationships more explicitly. Doing so would enhance the conceptual contribution of the study without requiring additional analyses.

Response: According to the suggestion, we have revised the opening paragraph of the Discussion to emphasize the dynamic interrelationships among social norms, sexual schemas, and sexual agency. We now explicitly describe how participants' accounts suggest that social norms shape sexual schemas, which may in turn influence the development and expression of sexual agency, thereby contributing to experiences of sexual satisfaction. This revision clarifies the interplay among the three categories while remaining faithful to the qualitative and interpretive nature of our findings. **[Page 15, paragraph 2]**

Comment 3: The manuscript now clearly reports that both individual and couple interviews were conducted, which improves transparency. However, I encourage the authors to discuss more explicitly how these two interview formats may have influenced data generation and interpretation. Interviewing partners together may affect disclosure, agreement, or the co-construction of narratives, and a brief reflection on this issue would further strengthen the methodological discussion.

Response: According to the suggestion we have revised the Data Collection section to clarify the complementary role of the two interview formats. Specifically, we now explain that each participant was first interviewed individually, allowing sensitive experiences to be discussed in a private setting, followed by a dyadic interview that provided additional insights into shared experiences, relational dynamics, and the ways couples jointly negotiated meanings. We also acknowledge that the presence of a partner during dyadic interviews may have influenced participants' disclosure or expressions of disagreement and have clarified that these potential

influences were taken into account during data interpretation by comparing the individual and dyadic interview data. **[page 6, paragraph 3]**

Comment 4: The Discussion has been considerably improved. Nevertheless, it could benefit from a slightly broader comparison with findings from other sociocultural contexts. Such comparisons would help readers better distinguish which findings appear specific to the Iranian context and which may reflect broader psychosocial processes related to sexual satisfaction.

Response: We have expanded the Discussion to provide a broader cross-cultural perspective by clarifying which aspects of our findings appear to be more salient in conservative sociocultural contexts, such as Iran, and which reflect broader psychosocial processes that have also been observed across diverse cultural settings. [page 18, last paragraph, **[page 16 last paragraph, page 18]**
