# Supplementary material for: A qualitative exploration of the psychosocial factors of sexual satisfaction in Iranian newlyweds
Source: PLoS One. 2026 Aug 13;21(8):e0356277. doi: 10.1371/journal.pone.0356277 (PMC13472429; doi:10.1371/journal.pone.0356277)
Supplement: Peer review history

# Peer review: Decision Letter 0

**DOI:** 10.1371/journal.pone.0356277.r001

**Authors:** Shadab Shahali

2 Jun 2026

Dear Dr. Kazemi,

Thank you for submitting your manuscript to PLOS ONE. After careful consideration, we feel that it has merit but does not fully meet PLOS ONE’s publication criteria as it currently stands. Therefore, we invite you to submit a revised version of the manuscript that addresses the points raised during the review process.

Please submit your revised manuscript by Jul 18 2026 11:59PM. If you will need more time than this to complete your revisions, please reply to this message or contact the journal office at plosone@plos.org. When you're ready to submit your revision, log on to https://www.editorialmanager.com/pone/ and select the 'Submissions Needing Revision' folder to locate your manuscript file.

- A letter that responds to each point raised by the academic editor and reviewer(s). You should upload this letter as a separate file labeled 'Response to Reviewers'.
- A marked-up copy of your manuscript that highlights changes made to the original version. You should upload this as a separate file labeled 'Revised Manuscript with Track Changes'.
- An unmarked version of your revised paper without tracked changes. You should upload this as a separate file labeled 'Manuscript'.

If you would like to make changes to your financial disclosure, please include your updated statement in your cover letter. Guidelines for resubmitting your figure files are available below the reviewer comments at the end of this letter.

If applicable, we recommend that you deposit your laboratory protocols in protocols.io to enhance the reproducibility of your results. Protocols.io assigns your protocol its own identifier (DOI) so that it can be cited independently in the future. For instructions see: https://journals.plos.org/plosone/s/submission-guidelines#loc-laboratory-protocols. Additionally, PLOS ONE offers an option for publishing peer-reviewed Lab Protocol articles, which describe protocols hosted on protocols.io. Read more information on sharing protocols at https://plos.org/protocols?utm_medium=editorial-email&utm_source=authorletters&utm_campaign=protocols.

As the corresponding author, your ORCID iD is verified in the submission system and will appear in the published article. PLOS supports the use of ORCID, and we encourage all coauthors to register for an ORCID iD and use it as well. Please encourage your coauthors to verify their ORCID iD within the submission system before final acceptance, as unverified ORCID iDs will not appear in the published article. *Only*  the individual author can complete the verification step; PLOS staff *cannot*  verify ORCID iDs on behalf of authors.

We look forward to receiving your revised manuscript.

Kind regards,

Shadab Shahali, PHD

Academic Editor

PLOS One

Journal requirements:

When submitting your revision, we need you to address these additional requirements.

1. Please ensure that your manuscript meets PLOS ONE's style requirements, including those for file naming. The PLOS ONE style templates can be found at

https://journals.plos.org/plosone/s/file?id=wjVg/PLOSOne_formatting_sample_main_body.pdf and

https://journals.plos.org/plosone/s/file?id=ba62/PLOSOne_formatting_sample_title_authors_affiliations.pdf

2. In the online submission form you indicate that your data is not available for proprietary reasons and have provided a contact point for accessing this data. Please note that your current contact point is a co-author on this manuscript. According to our Data Policy, the contact point must not be an author on the manuscript and must be an institutional contact, ideally not an individual. Please revise your data statement to a non-author institutional point of contact, such as a data access or ethics committee, and send this to us via return email. Please also include contact information for the third party organization, and please include the full citation of where the data can be found.

If the reviewer comments include a recommendation to cite specific previously published works, please review and evaluate these publications to determine whether they are relevant and should be cited. There is no requirement to cite these works unless the editor has indicated otherwise.

[Note: HTML markup is below. Please do not edit.]

Reviewers' comments:

Reviewer's Responses to Questions

**Comments to the Author**

1. Is the manuscript technically sound, and do the data support the conclusions?

Reviewer #1: Partly

Reviewer #2: Yes

**********

2. Has the statistical analysis been performed appropriately and rigorously?

Reviewer #1: N/A

Reviewer #2: N/A

**********

3. Have the authors made all data underlying the findings in their manuscript fully available?

The PLOS Data policy

Reviewer #1: No

Reviewer #2: Yes

**********

4. Is the manuscript presented in an intelligible fashion and written in standard English?

Reviewer #1: No

Reviewer #2: No

**********

Reviewer #1: The manuscript addresses an important and understudied topic and presents rich qualitative data that provide valuable insight into the lived experiences of Iranian newlyweds regarding sexuality, relationships, gender expectations, and social norms. The qualitative approach is generally appropriate for the research aims, and the findings have the potential to make a meaningful contribution to the literature on sexual satisfaction within understudied sociocultural contexts. However, I selected “Partly” regarding technical soundness because substantial revisions are needed to improve conceptual clarity, methodological transparency, thematic organization, and alignment between some thematic labels and the presented qualitative data. In addition, while this is a qualitative study and statistical analysis is not applicable, I selected “No” regarding data availability because the current Data Availability Statement may not fully align with PLOS ONE data-sharing requirements and would benefit from clarification regarding the accessibility of the qualitative data and the ethical restrictions surrounding data sharing. I also selected “No” regarding language and presentation because the manuscript contains numerous grammatical, stylistic, formatting, and organizational issues that substantially affect readability and clarity.

Please see the attached document for detailed, section-specific reviewer comments and suggestions.

Reviewer #2: It is a good study that is done within a specific social context. Some of the findings may be repetitive and nothing new, but the study in this specific field is valuable. Overall, it is publishable with the changes highlighted.

**********

what does this mean?). If published, this will include your full peer review and any attached files.

If you choose “no”, your identity will remain anonymous but your review may still be made public.

**Do you want your identity to be public for this peer review?**  For information about this choice, including consent withdrawal, please see our Privacy Policy

Reviewer #1: **Yes:**  Daisy Z. Hu

Reviewer #2: **Yes:**  Alireza Nikbakht Nasrabadi

**********

[NOTE: If reviewer comments were submitted as an attachment file, they will be attached to this email and accessible via the submission site. Please log into your account, locate the manuscript record, and check for the action link "View Attachments". If this link does not appear, there are no attachment files.]

To ensure your figures meet our technical requirements, please review our figure guidelines: https://journals.plos.org/plosone/s/figures

You may also use PLOS’s free figure tool, NAAS, to help you prepare publication quality figures: https://journals.plos.org/plosone/s/figures#loc-tools-for-figure-preparation.

NAAS will assess whether your figures meet our technical requirements by comparing each figure against our figure specifications.

Attachment Submitted filename: Reviewer_Comments_PONE_26_20783.docx.docx

Attachment Submitted filename: PONE-D-26-20783 RVZD11.pdf

# Author comment: Author response to Decision Letter 1

**DOI:** 10.1371/journal.pone.0356277.r002

20 Jun 2026

Dear Editor and Reviewers

We sincerely thank the reviewers for the thoughtful, constructive, and valuable comments on our manuscript. We highly appreciate the reviewers’ careful evaluation and insightful suggestions, which have helped us improve the quality of the manuscript. We have carefully addressed all comments and, to the best of our ability, incorporated the suggested revisions throughout the manuscript. We hope that the revisions made adequately respond to the reviewer’s concerns and that the revised version will meet with the reviewer’s approval. The manuscript has been revised according to the suggestions. The point-by-point response to the comments have been presented as follows:

Reviewers' comments:

Reviewer #1:

This study explores the psychosocial factors related to sexual satisfaction among Iranian newlyweds using a qualitative approach. The topic is important and understudied, particularly within the Iranian sociocultural context, and the manuscript presents rich qualitative data that provide valuable insight into the lived experiences of participants regarding sexuality, relationships, gender expectations, and social norms. The study also addresses a sensitive topic that is often difficult to investigate in more conservative cultural settings, which adds to its potential contribution to the literature.

However, the manuscript would benefit from substantial revision to improve conceptual clarity, logical organization, methodological transparency, and writing quality. In particular, the Introduction and Discussion sections would benefit from clearer structure and stronger integration with the broader literature, while the Methods section would benefit from clearer organization of procedures, participant structure, and analytic descriptions. Several thematic labels in the Results section could also be refined for greater conceptual precision and alignment with the qualitative data. Additionally, the manuscript requires considerable editing for grammar, formatting, and overall readability in order to align more closely with PLOS ONE reporting and formatting standards. Below, I provide detailed comments organized by manuscript section.

Abstract

Comment: The abstract repeatedly refers to “psychosocial factors,” but the term is not clearly defined. Because “psychosocial factors” can encompass a wide range of constructs depending on the context, I recommend briefly clarifying the term at its first mention by providing a few examples (e.g., cultural norms, sexual communication, gender expectations, or interpersonal dynamics related to sexual issues). This would improve conceptual clarity for readers unfamiliar with the topic area.

Response: The comment has been done [Abstract: Background; page 2]

Comment The following sentence is difficult to follow due to awkward sentence structure: “This qualitative study was conducted with a qualitative content analysis approach on 31 heterosexual newlyweds that less than two years had passed since started having sex.” I recommend revising this sentence for clarity and readability. For example, the authors could more clearly state that the study involved 31 heterosexual newlyweds who had been sexually active for less than two years.

Response: The sentence has been changed to: This qualitative study was conducted with a qualitative content analysis approach. The study involved 31 heterosexual newlyweds who had been sexually active for less than two years. [Abstract: Method; page 2]

Comment: In the Methods portion of the abstract, some methodological details may be overly technical for an abstract format. Specifically, the statement: “The criterion of data saturation was that no new inferential codes emerged after being repeated at least five times” introduces terminology (“inferential codes”) that is not explained and may not be necessary at the abstract level. This detail may be better reserved for the full Methods section.

Response: The sentence “The criterion of data saturation was that no new inferential codes emerged after being repeated at least five times.” has been omitted.

Comment Similarly, the sentence “The collected data were analyzed using Graneheim and Lundman method” may benefit from either a brief clarification of the analytic approach or simpler wording. If space is limited, the authors could instead state that the data were analyzed using qualitative content analysis.

Response: The comment has been done. [Abstract, method, page 2]

Comment In the Results section of the abstract, it would improve clarity to specify what the “three main categories” represent. For example, the authors could indicate that these were the three main psychosocial themes or categories identified through the qualitative analysis.

Response: The sentence change to: “Social norms of sexual issues, sexual schemas, and sexual agency. were the three main psychosocial categories identified through the qualitative analysis.” [Abstract, results, page 2]

Comment The Conclusion section would benefit from both grammatical revision and a clearer synthesis of the study’s findings and implications. The sentence beginning with “The results indicated that a more accurate understanding…” appears incomplete and difficult to follow. More importantly, the conclusion does not sufficiently summarize the key findings identified in the study, despite the manuscript having already outlined several important psychosocial themes (e.g., social norms, sexual schemas, and sexual agency). Rather than reiterating that “a more accurate understanding” is needed, the conclusion could more clearly state how the present findings contribute to that understanding and how these identified themes may inform culturally sensitive sexual education, counseling, or intervention programs for newlyweds in Iran.

Response: The conclusion section changes to “The findings provide insight into the key psychosocial dimensions underlying sexual satisfaction among newly married couples in Iran. These findings provide a culturally grounded understanding of how social norms, sexual schemas, and sexual agency shape sexual experiences and may inform the development of culturally sensitive sexual education, counseling, and intervention programs aimed at promoting sexual well-being and marital adjustment.” [Abstract, Cinclusion, page 2]

Introduction

Comment: The Introduction would benefit from a clearer conceptual structure and more coherent progression of ideas. At present, several paragraphs transition abruptly between individual, relational, and societal consequences of sexual satisfaction/dissatisfaction without sufficient conceptual linkage, making the overall argument difficult to follow.

In the opening paragraph, the manuscript states that “sexual satisfaction is a significant component of sexual health,” but does not define sexual satisfaction itself. I recommend briefly defining how sexual satisfaction is conceptualized within the literature before discussing its importance. This would provide readers with a clearer conceptual foundation for the study.

Response: The comment has been done. [Introduction, the first Paragraph, Page 3]

Comment: The statement “the lack of sexual satisfaction threatens society’s health” appears overly broad and insufficiently supported. The manuscript transitions very quickly from individual marital outcomes to societal-level consequences without adequately explaining the connection or providing supporting citations. I recommend either substantially revising this statement to make the argument more precise and evidence-based or narrowing the discussion to more directly established relational and psychological outcomes.

Response: The sentence” However, the lack of sexual satisfaction threatens society’s health by generating a complex network of social, physical, and psychological complications. “has been omitted. [Introduction, the first Paragraph, Page 3]

Comment: The first two paragraphs of the Introduction would benefit from clearer differentiation between sexual satisfaction and marital satisfaction, as the manuscript appears to move between these constructs interchangeably at several points. Because these are related but distinct constructs, the relationships between them should be explained more explicitly and consistently throughout the Introduction.

The discussion of consequences also shifts abruptly across levels of analysis (e.g., individual psychological outcomes, marital outcomes, and broader social implications) without clear transitions. For example, the manuscript moves from depression and self-esteem, to “social difficulties,” and then back to emotional divorce within the same section. Reorganizing these arguments and grouping consequences more coherently would substantially improve readability and conceptual clarity.

Response: The introduction reorganized according to the comment. [Introduction, Page 3-4]

Comment: The Introduction would benefit from a clearer concluding rationale explaining why studying sexual satisfaction is important, particularly in newlywed couples and within the Iranian sociocultural context. Currently, the early paragraphs discuss various consequences but do not clearly synthesize why this issue warrants the present investigation.

The paragraph beginning “The adverse and significant effects of sexual dissatisfaction…” relies heavily on a single cited study regarding early marriage and marital satisfaction. I recommend incorporating a broader and more diverse body of literature, including studies from multiple cultural and national contexts, to better establish the importance of sexual satisfaction during the early years of marriage. Additionally, the direction and nature of these associations should be described more explicitly (e.g., whether greater sexual satisfaction is associated with higher marital satisfaction, relationship stability, or quality of life).

Response: The sentences “Decreased marital satisfaction can increase the probability of depression [3], and decreased self-esteem [4], and sexual dysfunction” have been omitted.

Comment: The paragraph discussing “the cycle of sexual response” and environmental factors appears conceptually disconnected from the surrounding discussion. The transition from physiological sexual response, to sexual pleasure, to environmental influences and biopsychosocial framing is difficult to follow. I recommend either substantially revising this paragraph for clarity or integrating the biopsychosocial framework more naturally into the broader literature review.

Response: The paragraph “Although the cycle of sexual response, followed by sexual pleasure, goes through a physiological …… information” has been deleted.

Comment: More generally, the Introduction could be strengthened by improving the transition into the Iranian sociocultural context. Rather than introducing the Iranian context somewhat abruptly, the authors may consider first summarizing broader international evidence regarding sexual satisfaction and marital outcomes, and then explaining why the Iranian context may differ due to distinct sociocultural norms surrounding sexuality, gender, and marriage.

The discussion of Iranian sociocultural factors would benefit from greater specificity and clearer explanation. For example, the manuscript states that “sociocultural constraints and inadequate knowledge are key challenges,” but does not sufficiently explain what these constraints are or how they influence sexual satisfaction and communication among newlyweds.

Response: The comment has been done. [Introduction, the first Paragraph, Page 4]

Comment: Several sentences in the Introduction are conceptually unclear and would benefit from revision for clarity and precision. For example, the phrase “the development of sexual concepts as a cognitive generation about sexual aspect” is difficult to interpret. Similarly, statements regarding women’s and men’s attitudes being formed in a “sociocultural context, which has a dissimilar view on sexual issues” are vague and should more explicitly describe the relevant cultural differences or expectations being referenced.

Response: Due to the opinion of the respected referee and one of the referees regarding the length of the introductory sentence " Compared to modern countries, in traditional societies such as Iran, the development of ….. dissimilar view on sexual issues. " has been deleted. [Introduction, the first Paragraph, Page 3]

Comment: The rationale for conducting the study in Iran is currently spread across multiple paragraphs with overlapping ideas, resulting in some redundancy and reduced clarity. Consolidating and reorganizing these sections would strengthen the overall narrative and improve the logical flow of the Introduction.

Response: The comment has been done. [Introduction, the first Paragraph, Page 3-4]

Comment: The justification for the qualitative design and the focus on newlyweds could also be integrated more cohesively. For example, the authors could first explain why the early years of marriage represent a particularly formative phase for sexual and relational development, then argue that the limited existing evidence and sociocultural specificity of the topic make a qualitative approach especially appropriate for capturing lived experiences and identifying emergent psychosocial themes.

The final research question is generally appropriate and relevant to the aims of the study. However, the preceding rationale could more clearly build toward this question through a more logically organized review of the literature and clearer articulation of the study gap.

Response: The comment has been done [Introduction, the first Paragraph, Page 4]

Methods

Comment: The “Researcher Characteristics and Reflexivity” section appears closer to a positionality or reflexivity statement than a traditional methodological subsection. While reflexivity is important in qualitative research, the authors should ensure that this section aligns with the journal’s reporting standards and qualitative reporting guidelines.

Response: The subsection “Researcher Characteristics and Reflexivity” has been omitted and the comment has been done. [Page 5-7, Method]. Also, some unnecessary reports have been omitted.

Comment: The participant description would benefit from clearer demographic reporting. Although participant-level information is later presented in Table 1, the main text should also summarize key demographic characteristics such as age ranges or mean ages, educational background, and other relevant sample characteristics. In addition, the manuscript does not clarify whether participants were ethnically homogeneous or whether there was diversity in ethnicity or cultural background, which may be relevant to interpretation of the findings.

Response: The comments have been done. [Page 5; method/Participants subsection]

Comment: The inclusion and exclusion criteria require clarification. Specifically, the sentence regarding pregnancy and infertility is difficult to follow, and it is unclear whether pregnancy and infertility were treated as exclusion criteria because of their potential influence on sexual satisfaction. This should be stated more explicitly.

Response: The comments have been done. [Page 5; method/Participants subsection]

Comment: The description of data saturation remains somewhat unclear. The statement that “no new inferential codes emerged after being repeated at least five times” would benefit from additional clarification or simpler wording, as the concept of “inferential codes” is not sufficiently explained for readers unfamiliar with the analytic framework.

Response: The sentence “Data collection continued until no new data emerged.” has been replaced.

Comment: The manuscript states that purposive sampling aimed to maximize diversity in age, education, and duration of marriage/sexual activity, and these characteristics are shown in Table 1. However, the text does not explicitly direct readers to the table or summarize these characteristics in the narrative. Including brief summary statistics and clearer reference to the table would improve readability and transparency.

Response: The comment has been done. [Method/ page 5]

Comment: The overall structure and chronology of the “Procedure and Data Collection”

Attachment Submitted filename: Response to Reviewers.pdf

# Peer review: Decision Letter 1

**DOI:** 10.1371/journal.pone.0356277.r003

**Authors:** Shadab Shahali

13 Jul 2026

Dear Dr. Ashraf Kazemi,

Thank you for submitting your manuscript to PLOS ONE. After careful consideration, we feel that it has merit but does not fully meet PLOS ONE’s publication criteria as it currently stands. Therefore, we invite you to submit a revised version of the manuscript that addresses the points raised during the review process.

Please submit your revised manuscript by  Aug 12 2026 11:59PM. If you will need more time than this to complete your revisions, please reply to this message or contact the journal office at plosone@plos.org. When you're ready to submit your revision, log on to https://www.editorialmanager.com/pone/ and select the 'Submissions Needing Revision' folder to locate your manuscript file.

- A letter that responds to each point raised by the academic editor and reviewer(s). You should upload this letter as a separate file labeled 'Response to Reviewers'.
- A marked-up copy of your manuscript that highlights changes made to the original version. You should upload this as a separate file labeled 'Revised Manuscript with Track Changes'.
- An unmarked version of your revised paper without tracked changes. You should upload this as a separate file labeled 'Manuscript'.

If you would like to make changes to your financial disclosure, please include your updated statement in your cover letter. Guidelines for resubmitting your figure files are available below the reviewer comments at the end of this letter.

If applicable, we recommend that you deposit your laboratory protocols in protocols.io to enhance the reproducibility of your results. Protocols.io assigns your protocol its own identifier (DOI) so that it can be cited independently in the future. For instructions see: https://journals.plos.org/plosone/s/submission-guidelines#loc-laboratory-protocols. Additionally, PLOS ONE offers an option for publishing peer-reviewed Lab Protocol articles, which describe protocols hosted on protocols.io. Read more information on sharing protocols at https://plos.org/protocols?utm_medium=editorial-email&utm_source=authorletters&utm_campaign=protocols.

As the corresponding author, your ORCID iD is verified in the submission system and will appear in the published article. PLOS supports the use of ORCID, and we encourage all coauthors to register for an ORCID iD and use it as well. Please encourage your coauthors to verify their ORCID iD within the submission system before final acceptance, as unverified ORCID iDs will not appear in the published article. *Only*  the individual author can complete the verification step; PLOS staff *cannot*  verify ORCID iDs on behalf of authors.

We look forward to receiving your revised manuscript.

Kind regards,

Shadab Shahali, PHD

Academic Editor

PLOS One

Journal Requirements:

If the reviewer comments include a recommendation to cite specific previously published works, please review and evaluate these publications to determine whether they are relevant and should be cited. There is no requirement to cite these works unless the editor has indicated otherwise.

Please review your reference list to ensure that it is complete and correct. If you have cited papers that have been retracted, please include the rationale for doing so in the manuscript text, or remove these references and replace them with relevant current references. Any changes to the reference list should be mentioned in the rebuttal letter that accompanies your revised manuscript. If you need to cite a retracted article, indicate the article’s retracted status in the References list and also include a citation and full reference for the retraction notice.

[Note: HTML markup is below. Please do not edit.]

Reviewers' comments:

Reviewer's Responses to Questions

**Comments to the Author**

Reviewer #3: (No Response)

Reviewer #4: All comments have been addressed

**********

2. Is the manuscript technically sound, and do the data support the conclusions?

Reviewer #3: Yes

Reviewer #4: Partly

**********

3. Has the statistical analysis been performed appropriately and rigorously?

Reviewer #3: N/A

Reviewer #4: N/A

**********

4. Have the authors made all data underlying the findings in their manuscript fully available?

The PLOS Data policy

Reviewer #3: No

Reviewer #4: Yes

**********

5. Is the manuscript presented in an intelligible fashion and written in standard English?

Reviewer #3: Yes

Reviewer #4: Yes

**********

Reviewer #3: This manuscript reports a qualitative content analysis study exploring psychosocial factors associated with sexual satisfaction among 31 Iranian newlyweds. The study addresses a genuine gap in the literature on sexual health in conservative cultural settings. The three-category framework on social norms, sexual schemas, and sexual agency is conceptually coherent and offers a culturally grounded perspective with meaningful implications for premarital counseling and sexual health education in Iran. The paper is broadly suitable for this journal's readership and scope. However, several presentation and framing issues require attention before acceptance. While the paper generally provides rich and interesting insights, the manuscript is still hard to follow in some places, as outlined below. Also, responses to the previous round of reviews are often very short and sometimes difficult to follow. Many sentences for which the reviewer (1) suggested improvements were simply removed without further explanation. Still, the revision based on the earlier reviews considerably improved the manuscript and, from my own reading of the manuscript, the manuscript could be accepted for publication in PLOS One given the minor/moderate points raised below were resolved.

I have to disclose that I myself have not used qualitative data analyses yet. Still, my review should be relevant as future readers of this article may similarly have only limited knowledge on qualitative data analyses. Accordingly, my review focusses on the introduction, results, and discussion, as well as on the clarity of the presentation overall.

Minor/moderate points:

- The manuscript would benefit from further language editing and proofreading (e.g. in the abstract: “Psychosocial factors related to sexual issues […] related to sexual issues”, “This qualitative study was conducted with a qualitative content analysis approach. […] The data were analyzed using qualitative content analysis.”; “interviews conducted either individually or in dyadic settings. conducted either individually or in dyadic settings”; results, “cods”, “One of the man participants”, “One of the woman participants”)

- Abstract, “Consolidated Criteria for Reporting Qualitative Research guidelines”: Please provide the mentioned guidelines (e.g. in a table) in the main text or supplement.

- Abstract, “The data were collected and analyzed through individual and joint semi-structured interviews.”, and paragraph headed “Participants” in the “Methods and Materials” section: As already mentioned in the previous reviews, the cited sentence is not entirely clear, as it could mean that all participants went through both individual and joint interviews. Instead, it should be clarified that part of the sample consisted of individuals interviewed without their partner, and also couples interviewed jointly, while also mentioning the exact numbers for both in the abstract. In the “Participants” paragraph, it should also be clarified that some were interviewed individually and others in a joint interview (while also mentioning the exact numbers of participants for both) – this information is provided later (at the end of the subsection “Procedure and Data collection”), but should be clear earlier.

- In the first sentences of the introduction, the focus is set to married couples and marital satisfaction only. However, the research questions and findings may equally apply to couples in a committed romantic relationship without being married. Hence, I would either explain the direct focus on married couples only, or start off a little broader considering couples in committed romantic relationships in general.

- In the introduction and in the discussion, biological variables are mentioned without further explanation or definition (“sexual satisfaction is a multidimensional construct that extends beyond biological functioning”, “sexual experiences were not merely individual or biological but socially constructed”, “Sexual education programs in conservative contexts such as Iran should go beyond biological information”). Please clarify what kind of biological factors are meant here, by shortly defining and possibly also explaining how they are relevant.

- Abstract, Methods: It is stated that "sampling was implemented using a purposeful method and continued until data saturation." However, there are no details on how saturation was assessed or at which point it was reached. Please provide a short description of the criterion used to determine saturation (e.g. no new codes emerging across successive interviews), and an indication of approximately when saturation was reached relative to the total sample. The paper also notes that "early data saturation among male participants" may have occurred due to cultural constraints on male disclosure — but if saturation was reached early for men, this raises a serious question about whether men's experiences are adequately represented in the findings. A sample of 12 men is already skewed relative to 19 women; if saturation was reached early due to underrepresentation of male perspectives rather than genuine thematic exhaustion, the findings may systematically underrepresent men's experiences, which should be discussed.

- Methods, Procedure and Data collection, “In order to prevent the bias of the spouse’s presence in couple interviews, an attempt was made to deepen the interviews with questions such as “What do you think of your spouse’s statements?””: The sentence does not quite make sense conceptually. Asking “What do you think of your spouse’s statements?” does not really prevent bias from the spouse’s presence. Instead, it draws attention to the spouse’s statements and may even increase social desirability or conformity pressure. Please clarify if this is a phrasing error in describing the procedure or whether the interviews were actually conducted this way. If the latter, potential implications for results from the interviews should be discussed critically.

- Results, “Of the 34 eligible individuals invited for the study, 31 individuals […]”: Please clarify what happened to the remaining 3 participants.

- Several category labels are questionable from my point of view. “Social norms of sexual issues” could rather be “Social norms surrounding premarital sexuality”, “Premarital sexual norms”, or “Social norms regulating premarital sexual behavior”. Instead of “Sexual qualification”, “Sexual self-presentation”, “Perceived sexual competence”, or “Sexual attractiveness and competence” may be more suitable. Instead of “Sexual agency”, please consider “Sexual agency and control” or “Sexual power and control”. Please consider changing the labels throughout the manuscript. Also, it appears the subcategory labels in the subsection “sexual agency” do not match (e.g. “sexual knowledge and skills” versus “sexual awareness and skill”).

- In some Discussion sections I would consider adding relevant citations to better connect this study’s findings with earlier research, for example in the paragraph beginning with “These psychological factors”.

- Discussion, “Consistent with social constructionist perspectives, sexual experiences were not merely individual or biological but socially constructed through dominant cultural narratives, religious traditions, and gendered expectations [22].”: Here it is unclear whether the finding (“sexual experiences were not merely individual or biological but socially constructed”) refers to this study or the citation (Burr, 2024) – please clarify; if the former please elaborate on what kind of individual, biological, and social factors are referred to here.

- The conclusion is still very brief without adding any integrative insights. Please enrich the conclusion by shortly stating what the three categories collectively reveal about sexual satisfaction as a socially mediated phenomenon in the Iranian context, potentially also stressing the single most important implication(s) for policy or practice.

Reviewer #4: General comment

I would like to thank the authors for their careful and thorough revision. The manuscript has improved substantially since the previous version. The Introduction is more coherent, the methodological reporting has been strengthened, the Discussion has been expanded, and the practical implications are now better articulated. Overall, I believe the manuscript now makes a valuable contribution to the literature on sexual satisfaction in the Iranian sociocultural context.

I have only a few minor comments that I believe would further strengthen the manuscript.

Comment 1: The three overarching categories ("Social norms of sexual issues", "Sexual schemas", and "Sexual agency") provide a clear and useful framework for organizing the findings. However, it remains unclear whether these categories were developed entirely inductively from the data or whether they were informed by existing theoretical concepts from the literature. Given that notions such as sexual agency and sexual schemas are already well established in sexuality research, I encourage the authors to clarify their analytical approach and explain more explicitly how these categories emerged during the analytic process.

Comment 2: One aspect that could further strengthen the manuscript would be to make the relationships between the three identified categories more explicit. Throughout the Discussion, the findings suggest that social norms shape sexual schemas, which in turn influence sexual agency and, ultimately, sexual satisfaction. Rather than presenting these categories primarily as parallel themes, the authors may wish to discuss their dynamic interrelationships more explicitly. Doing so would enhance the conceptual contribution of the study without requiring additional analyses.

Comment 3 : The manuscript now clearly reports that both individual and couple interviews were conducted, which improves transparency. However, I encourage the authors to discuss more explicitly how these two interview formats may have influenced data generation and interpretation. Interviewing partners together may affect disclosure, agreement, or the co-construction of narratives, and a brief reflection on this issue would further strengthen the methodological discussion.

Comment 4 : The Discussion has been considerably improved. Nevertheless, it could benefit from a slightly broader comparison with findings from other sociocultural contexts. Such comparisons would help readers better distinguish which findings appear specific to the Iranian context and which may reflect broader psychosocial processes related to sexual satisfaction.

**********

what does this mean?). If published, this will include your full peer review and any attached files.

If you choose “no”, your identity will remain anonymous but your review may still be made public.

**Do you want your identity to be public for this peer review?**  For information about this choice, including consent withdrawal, please see our Privacy Policy

Reviewer #3: No

Reviewer #4: No

**********

[NOTE: If reviewer comments were submitted as an attachment file, they will be attached to this email and accessible via the submission site. Please log into your account, locate the manuscript record, and check for the action link "View Attachments". If this link does not appear, there are no attachment files.]

To ensure your figures meet our technical requirements, please review our figure guidelines: https://journals.plos.org/plosone/s/figures

You may also use PLOS’s free figure tool, NAAS, to help you prepare publication quality figures: https://journals.plos.org/plosone/s/figures#loc-tools-for-figure-preparation.

NAAS will assess whether your figures meet our technical requirements by comparing each figure against our figure specifications.

# Author comment: Author response to Decision Letter 2

**DOI:** 10.1371/journal.pone.0356277.r004

20 Jul 2026

Dear Editor and Reviewers

We are thankful for the comprehensive review and insightful suggestions for the manuscript entitled “A qualitative exploration of the psychosocial factors of sexual satisfaction in Iranian newlyweds”. The manuscript has been revised according to the suggestions. The point-by-point response to the comments have been presented as follows:

Journal Requirements:

If the reviewer comments include a recommendation to cite specific previously published works, please review and evaluate these publications to determine whether they are relevant and should be cited. There is no requirement to cite these works unless the editor has indicated otherwise.

Please review your reference list to ensure that it is complete and correct. If you have cited papers that have been retracted, please include the rationale for doing so in the manuscript text, or remove these references and replace them with relevant current references. Any changes to the reference list should be mentioned in the rebuttal letter that accompanies your revised manuscript. If you need to cite a retracted article, indicate the article’s retracted status in the References list and also include a citation and full reference for the retraction notice.

Response: We clarify that the reference list has not been modified. In addition, we carefully re-checked all references to verify their retraction status. We confirm that none of the cited references has been retracted.

Comments to the Author

Reviewer #3: Minor/moderate points:

- The manuscript would benefit from further language editing and proofreading (e.g. in the abstract: “Psychosocial factors related to sexual issues […] related to sexual issues”, “This qualitative study was conducted with a qualitative content analysis approach. […] The data were analyzed using qualitative content analysis.”; “interviews conducted either individually or in dyadic settings. conducted either individually or in dyadic settings”; results, “cods”, “One of the man participants”, “One of the woman participants”)

Response: Thank you for your valuable comment. We carefully proofread the entire manuscript and revised the language to improve clarity, readability, and grammatical accuracy. Specifically, redundant wording and repeated phrases were removed, typographical errors (e.g., cods → codes) were corrected, and grammatically incorrect expressions. We also revised the abstract and other relevant sections to eliminate repetitive expressions. [Abstract, page 1; Results: page 9-11]

- Abstract, “Consolidated Criteria for Reporting Qualitative Research guidelines”: Please provide the mentioned guidelines (e.g. in a table) in the main text or supplement.

Response: We have revised the Methods section to explicitly state that the study was reported in accordance with the Consolidated Criteria for Reporting Qualitative Research (COREQ) guidelines. In addition, we have included the completed COREQ checklist as a supplementary file, indicating where each reporting item is addressed in the revised manuscript. [Sup 1]

- Abstract, “The data were collected and analyzed through individual and joint semi-structured interviews.”, and paragraph headed “Participants” in the “Methods and Materials” section: As already mentioned in the previous reviews, the cited sentence is not entirely clear, as it could mean that all participants went through both individual and joint interviews. Instead, it should be clarified that part of the sample consisted of individuals interviewed without their partner, and also couples interviewed jointly, while also mentioning the exact numbers for both in the abstract. In the “Participants” paragraph, it should also be clarified that some were interviewed individually and others in a joint interview (while also mentioning the exact numbers of participants for both) – this information is provided later (at the end of the subsection “Procedure and Data collection”), but should be clear earlier.

Response: We agree that the original wording could have led to the misunderstanding that all participants underwent both individual and joint interviews. To eliminate this ambiguity, we have revised the Abstract and the “Participants” subsection of the Methods to clearly specify the interview format and the exact numbers. Specifically, we clarified that a total of 36 semi-structured interviews were conducted, comprising 31 individual interviews (19 women and 12 men) and 5 dyadic interviews involving 5 couples (10 participants). We also clarified that the dyadic interviews were conducted with couples whose partners had previously participated in individual interviews. These revisions improve the clarity and transparency of the study design. [Abstract: page 2; Method: page 7, paragraph 3]

- In the first sentences of the introduction, the focus is set to married couples and marital satisfaction only. However, the research questions and findings may equally apply to couples in a committed romantic relationship without being married. Hence, I would either explain the direct focus on married couples only, or start off a little broader considering couples in committed romantic relationships in general.

Response: We appreciate the reviewer's suggestion. The focus on married couples was intentional because the study was conducted in a sociocultural context where intimate and sexual relationships are socially, legally, and religiously recognized only within marriage. Therefore, broadening the scope to include unmarried couples would not be consistent with the cultural and ethical context in which the study was designed and approved. To clarify this rationale, we have revised the Introduction by explicitly explaining that the importance of investigating sexual satisfaction among married couples should be understood within such sociocultural contexts. [Page 3, First paragraph]

- In the introduction and in the discussion, biological variables are mentioned without further explanation or definition (“sexual satisfaction is a multidimensional construct that extends beyond biological functioning”, “sexual experiences were not merely individual or biological but socially constructed”, “Sexual education programs in conservative contexts such as Iran should go beyond biological information”). Please clarify what kind of biological factors are meant here, by shortly defining and possibly also explaining how they are relevant.

Response: Accordingly, we have revised the relevant statements in the Introduction and Discussion by briefly defining biological factors as aspects of sexual functioning, including sexual desire, arousal, physiological sexual responses, and, where relevant, reproductive anatomy and physiology. [Page 3, paragraph 4]

- Abstract, Methods: It is stated that "sampling was implemented using a purposeful method and continued until data saturation." However, there are no details on how saturation was assessed or at which point it was reached. Please provide a short description of the criterion used to determine saturation (e.g. no new codes emerging across successive interviews), and an indication of approximately when saturation was reached relative to the total sample. The paper also notes that "early data saturation among male participants" may have occurred due to cultural constraints on male disclosure — but if saturation was reached early for men, this raises a serious question about whether men's experiences are adequately represented in the findings. A sample of 12 men is already skewed relative to 19 women; if saturation was reached early due to underrepresentation of male perspectives rather than genuine thematic exhaustion, the findings may systematically underrepresent men's experiences, which should be discussed.

Response: Methods section to provide additional details regarding both purposive sampling and data saturation. In the Participants subsection, we clarified how purposive sampling was implemented to recruit participants with diverse characteristics and experiences relevant to the study aim. In the Procedure and Data Collection subsection, we explained that data collection and analysis were conducted concurrently and that sampling continued until successive interviews yielded no new codes or concepts, indicating that data saturation had been achieved. Also, we agree that the original wording could have implied that early data saturation among male participants reflected comprehensive thematic coverage. To avoid this interpretation, we have revised the Limitations section to clarify that the apparent early saturation may have resulted from restricted disclosure due to sociocultural factors rather than complete exploration of men's experiences. We also explicitly acknowledge that this may have led to the underrepresentation of men's perspectives in the findings and that this limitation should be considered when interpreting the results. [Page 5: last line & Page 6 2 first lins]

- Methods, Procedure and Data collection, “In order to prevent the bias of the spouse’s presence in couple interviews, an attempt was made to deepen the interviews with questions such as “What do you think of your spouse’s statements?””: The sentence does not quite make sense conceptually. Asking “What do you think of your spouse’s statements?” does not really prevent bias from the spouse’s presence. Instead, it draws attention to the spouse’s statements and may even increase social desirability or conformity pressure. Please clarify if this is a phrasing error in describing the procedure or whether the interviews were actually conducted this way. If the latter, potential implications for results from the interviews should be discussed critically.

Response: This was a phrasing error in the description of the procedure rather than in the conduct of the interviews. Accordingly, we have revised the sentence to read: "Could you elaborate on what your spouse has just said?" to accurately reflect the follow-up question used during the dyadic interviews. [Page 7, Paragraph 2]

- Results, “Of the 34 eligible individuals invited for the study, 31 individuals […]”: Please clarify what happened to the remaining 3 participants.

Response: We have added this information to the Participants section and clarified that the remaining three eligible individuals declined to participate. [page 8, result, paragraph 4]

- Several category labels are questionable from my point of view. “Social norms of sexual issues” could rather be “Social norms surrounding premarital sexuality”, “Premarital sexual norms”, or “Social norms regulating premarital sexual behavior”. Instead of “Sexual qualification”, “Sexual self-presentation”, “Perceived sexual competence”, or “Sexual attractiveness and competence” may be more suitable. Instead of “Sexual agency”, please consider “Sexual agency and control” or “Sexual power and control”. Please consider changing the labels throughout the manuscript.

Response: We sincerely thank the reviewer for this thoughtful and constructive suggestion. The category of “Social norms of sexual issues” has been changed to “Premarital sexual norms”. Sexual qualification has been changed to “Perceived sexual competence”, Sexual agency” has been changed to “Sexual agency and control” [results section and table 3 and any related sentences in discussion]

-Also, it appears the subcategory labels in the subsection “sexual agency” do not match (e.g. “sexual knowledge and skills” versus “sexual awareness and skill”).

Response: We agree that consistency between category and subcategory labels is important. In our analysis, however, sexual knowledge and skills was conceptualized as a fundamental component of sexual agency rather than as an independent construct. Sexual agency reflects an individual's capacity to make informed decisions, communicate needs and preferences, and act intentionally in relation to sexuality. Such agency is inherently supported by adequate sexual knowledge and the skills required to understand, evaluate, and navigate sexual situations. Therefore, we believe that the subcategory is conceptually aligned with the overarching category, as it represents one of the essential psychological foundations through which sexual agency is developed and expressed. Nevertheless, we have carefully reviewed the wording throughout the manuscript to ensure greater consistency and clarity between the category and its subcategories.

- In some Discussion sections I would consider adding relevant citations to better connect this study’s findings with earlier research, for example in the paragraph beginning with “These psychological factors”.

Response: Our intention in this paragraph and others were to interpret the psychosocial factors identified in the present study rather than to make a general statement about psychosocial factors reported in the broader literature. To avoid this potential ambiguity, we have revised the wording to explicitly refer to the psychosocial factors identified in our study. [page 15: paragraph 3]

- Discussion, “Consistent with social constructionist perspectives, sexual experiences were not merely individual or biological but socially constructed through dominant cultural narratives, religious traditions, and gendered expectations [22].”: Here it is unclear whether the finding (“sexual experiences were not merely individual or biological but socially constructed”) refers to this study or the citation (Burr, 2024) – please clarify; if the former please elaborate on what kind of individual, biological, and social factors are referred to here.

Response: We agree that the original wording could be interpreted as referring either to the findings of the present study or to the cited literature. To improve clarity, we revised this section to explicitly distinguish between our interpretation of the findings and the existing theoretical perspective. Specifically, we introduced the social constructionist perspective as an established theoretical framework and clarified that the statement regarding the social construction of sexual experiences reflects the existing literature (Ref. 22), which provides a conceptual basis for interpreting our findings. This revision also improves the logical flow between the discussion of previous evidence (Ref. 21) and the theoretical explanation offered by social constructionism (Ref. 22) [page 15: last paragraph, page 16: first paragraph]

- The conclusion is still very brief without adding any integrative insights. Please enrich the conclusion by shortly stating what the three categories collectively reveal about sexual satisfaction as a socially mediated phenomenon in the Iranian context, potentially also stressing the single most important implication(s) for policy or practice.

Response: According to valuable suggestion, we have revised the conclusion to provide a more integrative interpretation of the three interrelated categories, emphasizing that sexual satisfaction among Iranian newlyweds is a socially mediated phenomenon shaped by the interplay of premarital sexual norms, sexual schemas, and sexual agency. We have also strengthened the practical implications by highlighting the importance of culturally sensitive sexual education, premarital counseling, and couple-based interventions that address restrictive social norms, promote healthy sexual schemas, and strengthen sexual agency. [Page 20, Conclusion section]

Reviewer #4: General comment

I would like to thank the authors for their careful and thorough revision. The manuscript has improved substantially since the previous version. The Introduction is more coherent, the methodological reporting has been strengthened, the Discussion has been expanded, and the practical implications are now better articulated. Overall, I believe the manuscript now makes a valuable contribution to the literature on sexual satisfaction in the Iranian sociocultural context.

I have only a few minor comments that I believe would further strengthen the manuscript.

Comment 1: The three overarching categories ("Social norms of sexual issues", "Sexual schemas", and "Sexual agency") provide a clear and useful framework for organizing the findings. However, it remains unclear whether these categories were developed entirely inductively from the data or whether they w

Attachment Submitted filename: Response_to_Reviewers_auresp_2.pdf

# Editor report: Decision Letter 2

**DOI:** 10.1371/journal.pone.0356277.r005

**Authors:** Shadab Shahali

2 Aug 2026

A qualitative exploration of the psychosocial factors of sexual satisfaction in Iranian newlyweds

PONE-D-26-20783R2

Dear Dr. Kazemi,

We’re pleased to inform you that your manuscript has been judged scientifically suitable for publication and will be formally accepted for publication once it meets all outstanding technical requirements.

Within one week, you’ll receive an e-mail detailing the required amendments. When these have been addressed, you’ll receive a formal acceptance letter and your manuscript will be scheduled for publication.

An invoice will be generated when your article is formally accepted. Please note, if your institution has a publishing partnership with PLOS and your article meets the relevant criteria, all or part of your publication costs will be covered. Please make sure your user information is up-to-date by logging into Editorial Manager at Editorial Manager® and clicking the ‘Update My Information' link at the top of the page. For questions related to billing, please contact billing support.

If your institution or institutions have a press office, please notify them about your upcoming paper to help maximize its impact. If they’ll be preparing press materials, please inform our press team as soon as possible -- no later than 48 hours after receiving the formal acceptance. Your manuscript will remain under strict press embargo until 2 pm Eastern Time on the date of publication. For more information, please contact onepress@plos.org.

Kind regards,

Shadab Shahali, PHD

Academic Editor

PLOS One

Additional Editor Comments (optional):

Reviewers' comments:

# Editor report: Acceptance letter

**DOI:** 10.1371/journal.pone.0356277.r006

**Authors:** Shadab Shahali

PONE-D-26-20783R2

PLOS One

Dear Dr. Kazemi,

I'm pleased to inform you that your manuscript has been deemed suitable for publication in PLOS One. Congratulations! Your manuscript is now being handed over to our production team.

At this stage, our production department will prepare your paper for publication. This includes ensuring the following:

* All references, tables, and figures are properly cited

* All relevant supporting information is included in the manuscript submission,

* There are no issues that prevent the paper from being properly typeset

You will receive further instructions from the production team, including instructions on how to review your proof when it is ready. Please keep in mind that we are working through a large volume of accepted articles, so please give us a few days to review your paper and let you know the next and final steps.

Lastly, if your institution or institutions have a press office, please let them know about your upcoming paper now to help maximize its impact. If they'll be preparing press materials, please inform our press team within the next 48 hours. Your manuscript will remain under strict press embargo until 2 pm Eastern Time on the date of publication. For more information, please contact onepress@plos.org.

You will receive an invoice from PLOS for your publication fee after your manuscript has reached the completed accept phase. If you receive an email requesting payment before acceptance or for any other service, this may be a phishing scheme. Learn how to identify phishing emails and protect your accounts at https://explore.plos.org/phishing.

If we can help with anything else, please email us at customercare@plos.org.

Thank you for submitting your work to PLOS ONE and supporting open access.

Kind regards,

PLOS ONE Editorial Office Staff

on behalf of

Dr. Shadab Shahali

Academic Editor

PLOS One
